# Synthesis and Preclinical Evaluation of Small-Molecule Prostate-Specific Membrane Antigen-Targeted Abiraterone Conjugate

**DOI:** 10.3390/molecules27248795

**Published:** 2022-12-12

**Authors:** Aleksei E. Machulkin, Ekaterina A. Nimenko, Nikolay U. Zyk, Anastasiia A. Uspenskaia, Galina B. Smirnova, Irina I. Khan, Vadim S. Pokrovsky, Alexander N. Vaneev, Roman V. Timoshenko, Vugara V. Mamed-Nabizade, Maria V. Zavertkina, Alexander Erofeev, Petr Gorelkin, Alexander G. Majouga, Nikolay V. Zyk, Elena S. Khazanova, Elena K. Beloglazkina

**Affiliations:** 1 Chemistry Department, Lomonosov Moscow State University, Leninskie Gory, Building 1/3, GSP-1, 119991 Moscow, Russia; 2 N.N. Blokhin Russian Cancer Research Center, 24 Kashirskoye sh., 115478 Moscow, Russia; 3 Biochemistry Department, RUDN University, Miklukho-Maklaya Str.6, 117198 Moscow, Russia; 4 Laboratory of Biophysics, National University of Science and Technology MISiS, 4 Leninskiy pr, 119049 Moscow, Russia; 5 Dmitry Mendeleev University of Chemical Technology of Russia, Miusskaya sq. 9, 125047 Moscow, Russia; 6 LLC Izvarino-Pharma, v. Vnukovskoe, Vnukovskoe sh., 5th km., Building 1, 108817 Moscow, Russia

**Keywords:** prostate cancer, abiraterone, drug delivery, prostate-specific membrane antigen

## Abstract

Prostate cancer is the second most common type of cancer among men. The main method of its treatment is androgen deprivation therapy, which has a wide range of side effects. One of the solutions to this challenge is the targeted delivery of drugs to prostate cancer cells. In this study, we performed the synthesis of a novel small-molecule PSMA-targeted conjugate based on abiraterone. Cytotoxicity, the induction of intracellular reactive oxygen species, and P450-cytochrome species inhibition were investigated for this conjugate PSMA-abiraterone. The conjugate demonstrated a preferential effect on prostate tumor cells, remaining inactive at up to 100 µM in human fibroblast cells. In addition, it revealed preferential efficacy, specifically on PSMA-expressing lines with a 65% tumor growth inhibition level on 22Rv1 (PSMA+) xenografts after 14-fold oral administration of **PSMA-Abi** at a single dose of 500 mg/kg (7.0 g/kg total dose) was observed. This compound showed significantly reduced acute toxicity with comparable efficacy compared to **AbiAc**.

## 1. Introduction

Prostate cancer is the second leading cancer of men across the globe, including the United States and Europe [1].

The treatment of advanced prostate cancer has been addressed by androgen deprivation, since the disease is directly dependent on androgens [2]. However, it is associated with adverse effects on bone tissue, metabolism, the cardiovascular system, and cognitive health [3]. Medical castration leads to a decrease in testosterone and dihydrotestosterone production by the testes, but the adrenal glands and even the cancerous prostate tissue continue to produce androgens, which ultimately leads to the continued growth of prostate cancer [4]. Currently, this stage continues to pose a problem for treatment. The addition of androgen antagonists is very effective in reducing prostate-specific membrane antigen (PSMA) expression, but this has very little effect on increasing longevity [5]. Several recent studies have continued to demonstrate the activation of the androgen receptor as a key factor in the continued growth of prostate cancer [6]. The pharmaceutical inhibition of androgen production has been shown to improve clinical outcomes [7].

A modern antiandrogen approved for use in clinical practice is abiraterone acetate (**AbiAc**) [8], which is a CYP17 inhibitor. In the 2012 COU-AA-302 study, the US FDA and the European Committee for Medicinal Products for Human Use (CHMP) recommended expanding the indications for abiraterone in patients with Castration-resistant prostate cancer (CRPC) with minimal clinical manifestations who had not previously received chemotherapy [9]. In the treatment of prostate-resistant prostate cancer, the drug was able to decrease the PSA level by more than 50% in 85% of patients who had not previously received chemotherapy, and in 33% of patients after ketoconazole therapy. In 40% of chemotherapy-naïve patients, the PSA level decreased by more than 90% [10]. **AbiAc** significantly reduces androgen production by blocking the cytochrome P450 17-alpha-hydroxylase (CYP17) enzyme. CYP17 is a cytochrome P450 enzyme located in the endoplasmic reticulum of the testes, ovaries, adrenal glands, and placenta. CYP17 controls the synthesis of glucocorticoids and sex hormones. The enzyme has both 17α-hydroxylase and C17, 20-lyase activity and plays a critical role in cortisol and androgen synthesis [11]. Thus, CYP17A1 inhibitors stop the proliferation of cancer cells by blocking this crucial enzyme for androgen biosynthesis. The absence of key androgens for tumor growth further induces apoptosis in the prostate gland [12].

Currently, there are several approaches used to enhance the pharmaceutical formulation of abiraterone in order to increase its activity and bioavailability, including KinetiSol^®^ [13] or delivery systems based on drug-nanoformulated forms [14,15]. An approach was also developed with the creation of a nanosized bioconjugate containing a second active component, survivin antibodies, in addition to abiraterone [16].

In fact, these approaches represent passive transport systems, while no studies on the active targeted delivery of abiraterone to prostate tissues have been presented to date. At the same time, this approach can significantly improve the tumor penetration of the conjugates created in this way, contributing to the improvement of the antitumor effect.

Low-molecular-weight antitumor agents can generally be divided into two categories: (1) nonspecific agents, which are commonly highly toxic with significant adverse effects, and (2) drugs that have their own tumor-specific targets and practically only interact with the target, having fewer non-specific adverse effects [17].

Active targeted delivery is most often used for the first group of antitumor drugs with pronounced toxicity (taxanes, dolastatin analogues, Vinca alkaloids, etc.) to deteriorate non-specific toxicity. Nevertheless, the active targeted delivery approach for an agent that is not the most highly toxic, such as abiraterone, is also possible. This approach can improve cancer-selectivity and, consequently, the efficacy of the drug. Accordingly, in this work, we focused on the creation of a conjugate for the targeted delivery of abiraterone based on PSMA (prostate-specific membrane antigen) ligands, which successfully proved themselves in the delivery of such therapeutic agents as docetaxel [18] and MMAE [19].

PSMA (glutamate carboxypeptidase 2, EC 3.4.17.21) is a highly specific membrane protein marker of prostate tumors. PSMA is expressed in normal, benign, and malignant prostate tissues, including intraepithelial neoplasia and metastatic specimens [20]. At the same time, its expression in normal prostate tissue is many times lower, in contrast to its neoplastic cells [21]. PSMA expression, however, is not limited to PCa or prostate tissue, as it is also expressed in the salivary glands, the duodenal epithelial brush border cells, the proximal tubule cells in the kidneys, a subpopulation of neuroendocrine cells in colonic crypts, and in the neovasculature of other solid tumors (such as neuroendocrine tumors, hepatocellular carcinoma, and thyroid cancer) [22,23,24,25].

This target has proven itself through numerous applications, for example, for the targeted delivery of fluorescent agents [26,27,28,29], therapeutic agents [30,31,32,33], and agents for photodynamic therapy [34,35,36]. The greatest success has been obtained for radiopharmaceutical conjugates—two of which have already been approved by the FDA [37].

Thus, the presented work is aimed at obtaining a conjugate of abiraterone with the PSMA ligand and its biological study.

## 2. Results and Discussion

### 2.1. Synthesis

To obtain the conjugate with abiraterone, the PSMA ligand **1** was used. Previously, it has demonstrated a high IC_50_ value of 9 ± 3 nM in comparison with 80 ± 3 nM for 2-PMPA [29]. Conjugates with the fluorescent agents Fam-5, SulfoCy5, and SulfoCy7 [29], as well as the therapeutic agents docetaxel [18] and MMAE [19], have also been previously obtained.

**Scheme 1:**
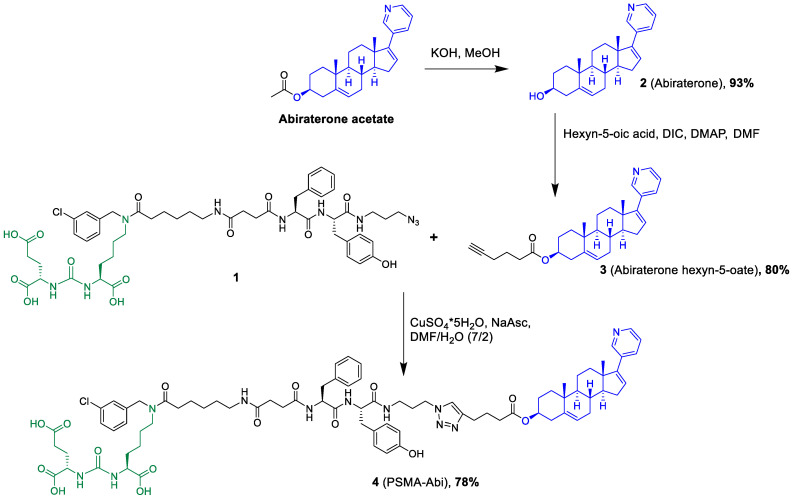
Synthesis of conjugate **PSMA-Abi** (**4**).

The synthesis of abiraterone **2** was carried out following the methodology in [38] with a number of modifications. The hydrolysis of ester groups was carried out in MeOH solution with KOH as a hydrolyzing agent. The reaction was controlled by TLC, and it was finished within 1.5 h from its initiation. The subsequent acylation required much greater efforts, and the reaction was only completed after 5 days from its start, which was probably due to the weak nucleophilic properties of the hydroxyl group of abiraterone. Nevertheless, compound **3** was obtained with a good yield of 80%. Then, it was introduced to a copper-mediated [3+2]-azide alkyne cycloaddition with PSMA-ligand **1**. As a result, conjugate **4 (PSMA-Abi)** was obtained with a 78% yield, which was then evaluated in in vitro and in vivo experiments (Scheme 1).

### 2.2. In Vitro Evaluation

#### 2.2.1. Evaluation of CC_50_ on Prostate Cancer Cell Lines 22Rv1, PC-3 and Human Adult Dermal Fibroblasts

PC-3 is the most common model of androgen-independent prostate cancer. Since this line is characterized by high malignancy and androgen resistance, PC-3 is an excellent experimental model with which to study new therapies for human prostate cancer [39]. A new prostate cancer cell line, 22Rv1, expresses PSMA and is sensitive to dihydrotestosterone; accordingly, this cell line is androgen-dependent, unlike PC-3 [40].

The in vitro efficacy study of the **PSMA-Abi** conjugate included an assessment of its cytotoxic potential towards prostate cancer cell cultures (Table 1). The achieved CC_50_ values for the **PSMA-Abi** (8.2 ± 3.1 μM) conjugate were three times lower than those for **AbiAc** (CC_50_ 22.7 ± 3.3 μM) in the 22Rv1 cell line. This conjugate demonstrated a statistically significant increase in cytotoxic potential compared to the parent compound. At the same time, the **PSMA-Abi** conjugate has better water solubility compared to the **AbiAc**, which fulfills the prerequisites for increasing the tumor-selectivity of the Abiraterone conjugate in in vivo experiments. The cytotoxicity of the conjugate towards the PC-3 cell line with low-to-negligible PSMA expression remains comparable to that of **AbiAc**. Thus, we observed an increase in the efficacy of the conjugate towards the PSMA-expressing line. Additionally, an evaluation in a non-tumor model was performed. The toxicity of both compounds was evaluated on human adult dermal fibroblasts (HDFa); both of the agents in the tested concentrations (0.4–100 µM) were non-toxic for human fibroblasts.

#### 2.2.2. Measurement of Reactive Oxygen Species Level

It is known that abiraterone affects redox homeostasis, leading to aberrant ROS production, which significantly increases the level of hydrogen peroxide in cells [42]. Therefore, for a preliminary assessment of PSMA-Abi’s effectiveness, we amperometrically measured the ROS levels in single cells (PC-3 and 22Rv1) using platinized nanoelectrodes [43,44].

**Figure 1:**
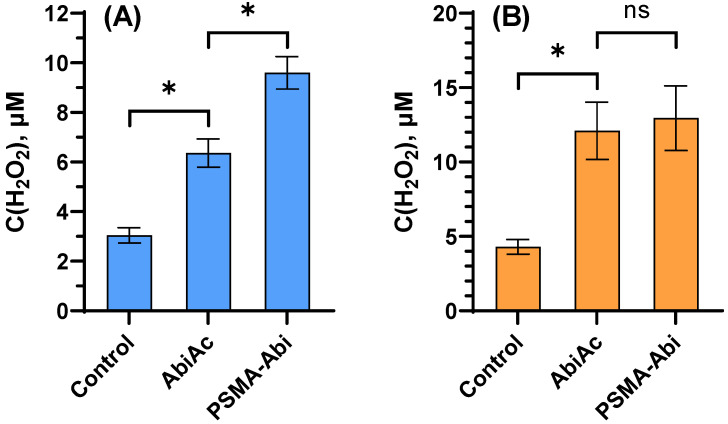
Comparison of the level of ROS/RNS inside the cells of 22Rv1 (**A**) and PC-3 (**B**) under the influence of **AbiAc** and **PSMA-Abi** (PSMA-Abiraterone). Cells were incubated for 1 h in RPMI-1640 (1% FBS) at 37 °C, 5% CO_2_ with anticancer drugs (IC_50_ concentration for each cell line). Results are shown as means ± SEM. *: *p* < 0.05 (one-way ANOVA).

In the experiment, **AbiAc** and a sample of **PSMA-Abi** conjugate that specifically binds to PSMA were used. The cell lines were incubated for 1 h at 37 °C with these preparations to allow the drugs to successfully penetrate the single cancer cells. In the case of **AbiAc**, after incubation for 1 h, the hydrogen peroxide level was significantly increased compared to the control, indicating cell apoptosis when exposed to Abiraterone, since ROS are the key inducers in this process. At the same time, the peroxide concentration following incubation with **PSMA-Abi** after 1 h remained at the level of the **AbiAc** sample towards the PC-3 cell line, but the ROS level in 22Rv1 showed a significant increase in comparison with both the control and **AbiAc** groups. Since the PC-3 cell line lacks PSMA on its membrane surface, the effect of the conjugates is not as significant as for the 22Rv1 cell line. Since this cell line has PSMA on its surface, the efficacy of the conjugate is greater than for **AbiAc**, as shown in Figure 1. Thus, **AbiAc** and **PSMA-Abi** have an effect on cell death in both cell lines. In the 22Rv1 cell line, the **PSMA-Abi** conjugate—due to its specificity to PSMA—causes significantly increased oxidative stress in cells, leading to further cell death.

#### 2.2.3. Cell Cycle Analysis

To compare the toxicity profile of both **PSMA-Abi** and **AbiAc** drugs, a cell cycle analysis was performed. Compared to the control cells, the percentage of 22Rv1 cells in the G2/M phase decreased as the concentration of **AbiAc** increased, whereas the level of G0/G1 cells increased, and the S cells were unaffected by the **AbiAc** treatment. The effect of **PSMA-Abi** was not statistically significant against a control group. However, the increase in 22Rv1 in G2/M phase was noticed at a concentration of IC_50_. It was recently demonstrated that cell cycle arrest is predominantly performed at G0/G1 for in 22Rv1 line after treatment by **AbiAc [45]**. When incubated with **AbiAc**, a dose-dependent correlation was observed regarding the increase in the cell fraction in the G0/G1 phase, reaching a maximum at IC_50_ in the concentration set studied (Figure S4 supplementary materials). At the same time, we observed practically unchanged number of cells in G0/G1 for **PSMA-Abi**. Simultaneously, the level of cells in this stage of the cell cycle remained comparable for these IC_50_ concentrations, which may indicate that the **PSMA-Abi** conjugate more effectively penetrates cells than **AbiAc** and transfers cells into the G0/G1 phase even at a concentration of 1/4 × IC_50._

### 2.3. In Vivo Evaluation

#### 2.3.1. Acute Toxicity

**Figure 2:**
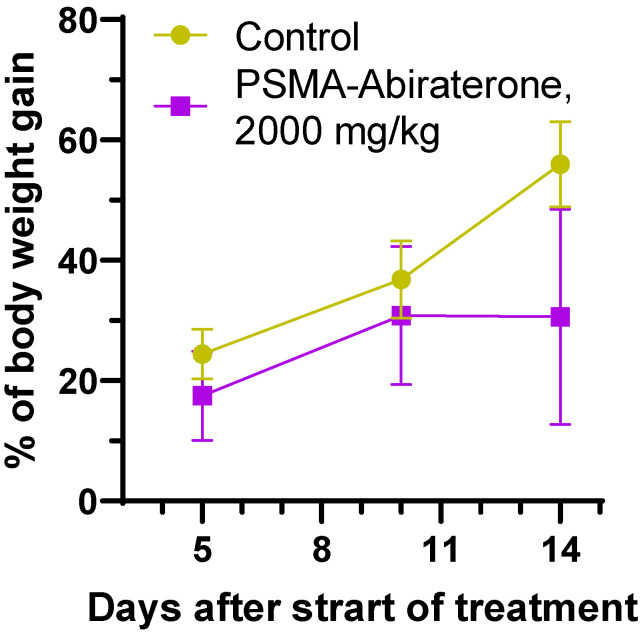
Body weight dynamics of mice after single-dose oral administration of **PSMA-Abi** (2000 mg/kg).

No animal death was observed at the dose of 2000 mg/kg when administered in a single dose. However, an analysis of their body weight dynamics compared to the control group showed a decrease in the growth rate in mice from 50 to 30% of their initial weight by the 14th day of administration (Figure 2). The dose of 2000 mg/kg is certainly a tolerable dose for mice. According to the data of the SDS manufacturer Janssen, there are data on the acute toxicity of the drug corresponding to LD_50_ = 800 mg/kg in mice. Nevertheless, compared with the available data, we see that while maintaining a comparable antitumor effect with the control drug, there is a significant decrease in toxicity, which, in the long term, allows us to expect fewer side effects from it [46]. In this case, it should be emphasized that the dosage of 2000 mg/kg does not lead to the death of any mice, only to a decrease in their body weight.

#### 2.3.2. Evaluation of Anticancer Effect

**Figure 3:**
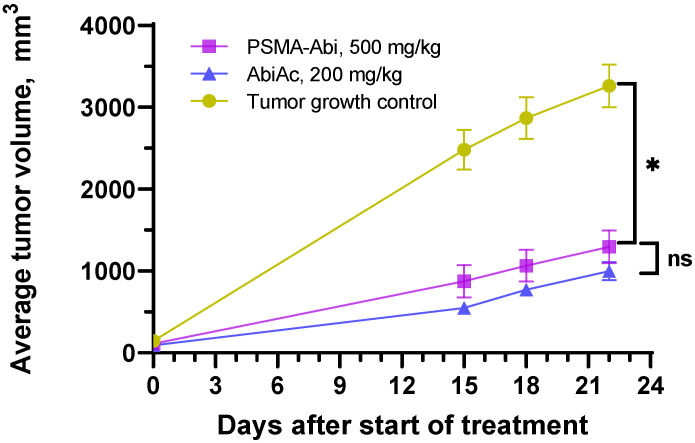
Growth dynamics of subcutaneous 22Rv1 prostate cancer xenografts under the effect of 14-fold oral administration of **PSMA-Abi** at a single concentration of 500 mg/kg/day (7.0 g/kg total) and **AbiAc** at 200 mg/kg/day (2.8 g/kg total).

The effect of the studied drugs was evaluated in terms of the inhibition of tumor growth. An in vivo comparative study showed a significant anti-tumor effect of both drugs—(TGI ≥ 60%): **PSMA-Abi**, TGImax = 65% (*p* = 0.0004, compared with the control group) and **AbiAc**: TGImax = 78% (*p* = 0.0001, compared with the control group)—after the end of the treatment. A comparative study showed no significant differences in efficacy in the two experimental groups. A prolonged retardation of tumor growth up to 8 days after the end of treatment was demonstrated with a preserved TGI of 60–65%. The 22Rv1 prostate cancer subcutaneous xenograft model was sensitive to both **PSMA-Abi** and **AbiAc** therapy (Figure 3). In both groups, the general condition and behavior of the mice during and after administration of the therapeutic agents were satisfactory without pathological changes or visible adverse effects. Thus, the results showed no significant difference in efficacy in both experimental groups. The tolerability of the treatment in the experimental groups was satisfactory.

#### 2.3.3. Pharmacokinetics

**Figure 4:**
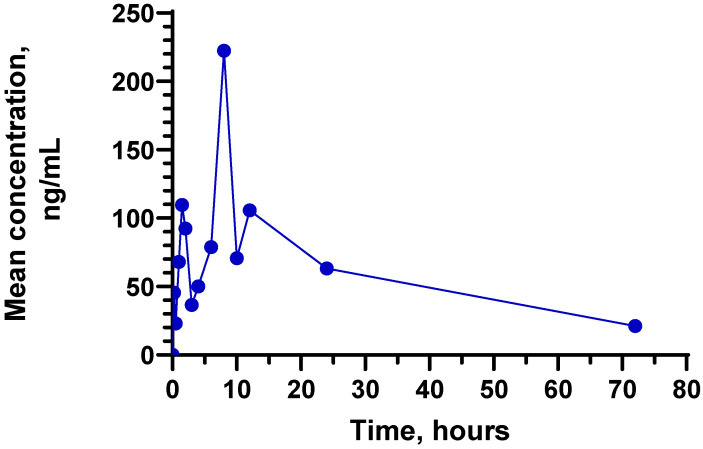
The mean pharmacokinetics curve of conjugate **PSMA-Abi** administered at a dose 1000 mg/kg to rats.

After the oral administration of **PSMA-Abi**, the maximum plasma concentrations of the drug in the laboratory animals were reached after 1.5 to 8 h and ranged from 190 to 537 ng/mL (Figure 4). The mean values of the areas under the pharmacokinetics curve, AUC_0–t_ and AUC_0–∞_, which characterize the bioavailability of **PSMA-Abi** after a single oral administration in rats, averaged 3791 ± 875 ng-h/mL and 4623 ± 1260 ng-h/mL. Plasma concentrations of the drug in rats were detected for at least 72 h following drug administration, with an average elimination half-life of 31.3 ± 8.7 h. (Table 2).

#### 2.3.4. Cytochrome Study

It is known that the efficacy and safety of drugs (drug candidates) generally depend on a drug’s concentration in the area of target molecules, which is most often related to the plasma concentration of drugs. In turn, the drug concentration in plasma depends on the processes of absorption, distribution, and elimination [47]. The elimination of drugs occurs via biotransformation (most often in the liver and/or intestinal mucosa) and/or excretion (most often by the kidneys through urine and/or liver through bile).

**Figure 5:**
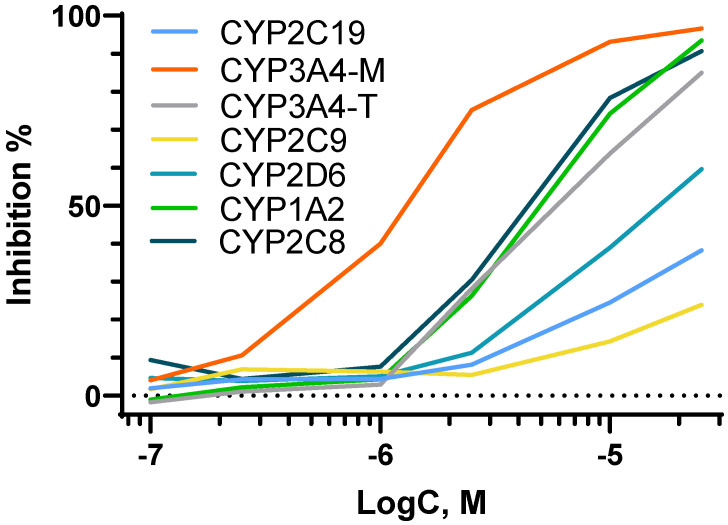
Inhibition of P450 cytochromes by **PSMA-Abi**.

Biotransformation in the liver occurs mainly with oxidation catalyzed by cytochrome P-450 isoenzymes (cytochrome P-450-dependent monooxygenase) in the endoplasmic reticulum of hepatocytes (biotransformation phase I), but it can also occur with the participation of enzyme systems not related to P-450, such as N-acetyl- and glucuronosPIyl-transferases (biotransformation phase II). Despite the diversity of cytochrome isoenzymes in the human body, drug biotransformation occurs with the participation of a predominantly limited number of members of the P-450 family. The most common of these are CYP1A2, CYP2C9, CYP2C19, CYP2D6, and the CYP3A4 isoenzyme, which also potentiate the oxidative biotransformation of many known drugs [48]. Thus, it is important that a newly developed drug does not have an inhibitory effect on this group of cytochrome isoenzymes. In this study, the activity of the main cytochrome P450 isoforms—1A2, 2C8, 2C9, 2C19, 2D6, and 3A4—was determined in the presence of **PSMA-Abi**. As a control, cytochrome activity was evaluated in the presence of known specific inhibitors. The inhibition curves are shown in Figure 5, and the IC_50_ values obtained are summarized in Table 3.

## 3. Conclusions

This work resulted in the synthesis of a low-molecular-weight conjugate with a highly selective PSMA ligand with abiraterone. When assessing the cytotoxicity of the conjugate **PSMA-Abi**, an increase in its cytotoxicity towards PSMA-expressing 22Rv1 cell lines, together with the absence of a significant change in the toxicity profile towards the PSMA-negative PC-3 cell line, when compared to **AbiAc** were found, with corresponding CC_50_ (**PSMA-Abi**) values of 8.2 and 8.1 µM. The effectiveness of the conjugate was evaluated in 22Rv1 xenograft murine models of prostate cancer. The conjugate **PSMA-Abi** demonstrated a 65% tumor growth inhibition level in the 22Rv1 (PSMA+) xenografts in comparison with 78% inhibition for **AbiAc** under the effect of a 14-fold oral administration of **PSMA-Abi** at a single dose of 500 mg/kg (7.0 g/kg total) and **AbiAc** at a single dose of 200 mg/kg (2.8 g/kg total). However, statistical analysis showed no significant differences in the inhibition of tumor growth in the two groups observed. Thus, the efficacies of both the conjugate and the reference sample were sufficiently high.

When attempting to evaluate acute toxicity, no specific LD_50_ value could be obtained due to the high dosage without animal death of 2000 mg/kg in the mice. Analysis of the body weight dynamics compared to the control group showed a decrease in the growth rate in mice from 50 to 30% of their initial weight by the 14th day of administration. Despite the decrease in the total body weights of the animals in the experiment, their conditions remained satisfactory. In comparison with the available data concerning the acute toxicity of **AbiAc**, we observed that while maintaining a comparable antitumor effect of **PSMA-Abi** in comparison with the control drug, there is a significant decrease in toxicity, which, in the long term, allows us to expect fewer side effects from it.

On this basis, the **PSMA-Abi** conjugate may be a promising candidate for further, expanded preclinical and clinical trials as an analogue for standard **AbiAc** formulation.

## 4. Experimental Section

### 4.1. Synthesis 

Conventional ^1^H and ^13^C NMR spectra were registered on a Bruker Avance 400 spectrometer (400 MHz for ^1^H and 101 MHz for ^13^C) in CDCl_3_ or DMSO-d6. Preparative column chromatography was performed on an INTERCHIM puriFlash 430. For purification and analysis of samples, we used a Shimadzu Prominence LC-20 system with a Phenomenex Luna C18 100A (150 × 4.6 mm) column in a column oven at 40 °C and a fraction collector coupled to a single quadrupole mass spectrometer Shimadzu LCMS-2020 with a dual DUIS-ESI-APCI ionization source. The mobile phases were A—0.1% formic acid in water, B—10 mM ammonium formate in water, and D—acetonitrile. The liquid chromatography (LC) parameters for analysis were a gradient flow of 1 mL/min (0–0.5 min with 5% D, 0.5–10.5 min with 5% to 100% D, 10.5–12 min with 100% D, and 12–14.5 min with 100% to 5% D) with optional UV detection for some compounds. The MS parameters were drying gas at 15.0 L/min, nebulizing gas at 1.5 L/min, desolvation line temperature of 250 °C, heat block temperature of 400 °C, interface voltage of −3.5 kV, and corona needle voltage of −3.5 kV. Positive ions (mass range 250–2000 Da, in some cases 155–2000 Da) and negative ions (mass range 100–2000 Da) were registered simultaneously. LC-MS method was used to determine the purity of synthesized ligands and conjugates; purity of all compounds investigated in vitro and/or in vivo were ≥95%. LCMS results were presented as three chromatograms: top—base peak chromatogram of the sample; middle—extracted-ion chromatogram of the targeted ion; bottom—base peak chromatogram of a blank sample.

High-resolution mass spectra (HR-MS) were registered on an Orbitrap Elite mass spectrometer (Thermo Scientific, USA) with an ESI ionization source. Compounds with a concentration of 0.1–10 µg/mL (in 1% formic acid in acetonitrile) were directly infused into the ion source with a syringe pump (5 µL/min). We did not use auxiliary and sheath gases. The spray voltage was ±3.5 kV, and the capillary temperature was set to 275 °C. The MS spectra were registered on an Orbitrap analyzer with a resolution of 480,000 (1 microscan; AGC target value of 1 × 10^6^; maximum inject time of 1000 ms, averaged from 10 spectra; and MS range 100–2000 Da, in some cases 200–4000 Da). We used DMSO and di-iso-octyl phthalate as internal calibration signals (*m*/*z* 157.03515 and 413.26623) in positive mode and dodecyl sulfate (*m*/*z* 265.14790) in negative mode.

### 4.2. Synthesis of Abiraterone

A total of 8.75 mL of 10% KOH solution was added to a solution of **AbiAc** (1.000 g; 2.55 mmol) in 12.5 mL of methanol. The mixture was stirred for 1.5 h. Reaction was monitored by TLC (MeOH:DCM, 1:50) according to the disappearance of the starting compound. Afterwards, the solvent was removed under reduced pressure. The resulting precipitate was dissolved in 40 mL of dichloromethane and 40 mL of water was added. The resulting mixture was stirred for one hour. Afterwards, the organic fraction was separated, and the solvent was removed under reduced pressure. The aqueous fraction was extracted with methylene chloride. The organic fraction was dried over Na_2_SO_4_. Afterwards, the solvent was removed under reduced pressure. The resulting compound was obtained with a yield of 93% (829 mg). ^1^H NMR (400 MHz, CDCl_3_, δ, ppm): 8.62 (m, 1H, Py), 8.46 (d, J = 4.7, 1H, Py), 7.64 (d, J = 7.65, 1H, Py), 7.22 (dd, J1 = 7.8, J2 = 4.8, 1H, Py), 5.99 (m, 1H, CH), 3.55 (m, 1H, OH), 2.23–2.35 (m, 3H, CH_2_), 2.03–2.10 (m, 3H, CH_2_), 1.44–1.90 (m, 11H, CH_2_), 1.08–1.13 (m, 1H, CH), 1.07 (s, 3H, CH_3_), 1.05 (s, 3H, CH_3_). ^13^C NMR (101 MHz, CDCl3, δ, ppm): 151.67 (Py), 147.88 (Py), 147.81 (C=C), 141.19 (C=C), 133.71 (Py), 132.98 (C=C), 129.26 (Py), 123.04 (C=C), 121.31 (Py), 71.64 (C-OH), 57.55 (CH), 50.35 (CH), 47.33 (CH), 42.31 (CH), 37.19 (CH_2_), 36.70 (CH_2_), 35.24 (CH_2_), 31.82 (CH_2_), 30.44 (CH), 20.87(CH_2_), 19.36 (CH_3_), and 16.59 (CH_3_).

### 4.3. Synthesis of Abiraterone Hexyn-5-oate

The following substances were added to a solution of abiraterone (1.139 g; 2.567 mmol) in 150 mL dichloromethane: hexyn-5-oic acid (0.345 g, 3.080 mmol, and 1.2 eq), DIC (0.388 g, 3.080 mmol, and 1.2 eq), and DMAP (0.094 g, 0.770 mmol, and 0.3 eq). Reaction was monitored by TLC (MeOH:DCM, 1:50) according to the disappearance of the starting compound. After 3 days, further amounts of hexyn-5-oic acid (0.104 g, 0.924 mmol, and 0.36 eq), DIC (0.117 g, 0.924 mmol, and 0.36 eq), and DMAP (0.113 g, 0.924 mmol, and 0.36 eq) were added. After stirring for an additional day, hexyn-5-oic acid (0.069 g, 0.616 mmol, and 0.24 eq), DIC (0.078 g, 0.616 mmol, and 0.24 eq), and DMAP (0.075 g, 0.616 mmol, and 0.24 eq) were added. The resulting mixture was stirred for another 24 h. The product was isolated by reverse column chromatography (Puriflash C18 HP 15 μ 35G, eluent: Water: acetonitrile, 40% to 50% of acetonitrile for 5 min; then, isocratic at 50% acetonitrile for 5 min, followed by 50% to 100% of acetonitrile for 5 min; finally, the column was washed with methanol for 10 min). The yield was 910 mg (80%). ^1^H NMR (400 MHz, DMSO-d6, δ, ppm): 8.62 (m, 1H, Py), 8.46 (d, J = 4.6, 1H, Py), 7.64 (d, J = 7,9, 1H, Py), 7.22 (dd, J1 = 7.8, J2 = 4.8, 1H, Py), 5.99 (s, 1H, CH), 5.42 (d, J = 4.8, 1H, CH), 4.59–4.67 (m, 1H, CH-O), 2.41–2.45 (m, 2H, CH_2_COO), 2.34–2.36 (m, 2H, CH_2_), 2.24–2.30 (m, 3H, CH_2_), 2.03–2.10 (m, 4H, CH_2_), 1.97–1.98 (m, 1H, CH), 1.83–1.89 (m, 3H, CH_2_), 1.45–1.81 (m, 8H, CH_2_), 1.11–1.20 (m, 1H, CH), 1.08 (s, 3H, CH_3_), 1.05 (s, 3H, CH_3_). ^13^C NMR (101 MHz, DMSO-d6, δ, ppm): 147.85 (Py), 147.19 (Py), 139.85 (Py), 133.32 (C=C), 129.05 (C=C), 123.43 (C=C), 121.98 (C=C), 83.67 (C≡C), 73.23(C≡C), 71.84 (O-CH), 57.00 (CH), 49.65 (CH), 46.66 (CH), 37.71(CH), 36.44 (CHa_2_), 36.32 (CH_2_), 32.60 (CH_2_), 30.97 (CH_2_), 29.89 (CH_3_), 27.37(CH_3_), 23.53 (CH_2_), 20.41 (CH_2_), 18.94 (CH_2_), 17.11 (CH_2_), and 16.28 (CH_2_). HRMS (ESI) *m/z* 444.2890 (calculated 444.2897 for C_30_H_37_NO_2_^+^ [M+H]^+^).

### 4.4. Synthesis of Conjugate PSMA-Abi

Compound **1** (2.498 g, 2.380 mmol, and 1 eq.) and Abiraterone-hexyn-5-oate **3** (1.108 g, 2.498 mmol, and 1.05 eq.) were dissolved in a mixture of 144 mL DMF and 48 mL of deionized water in a Schlenk flask. The flask was filled with argon, and then sodium ascorbate (0.551 g, 3.430 mmol, and 1.2 eq.) and copper sulfate pentahydrate (0.237 g, 1.143 mmol, and 0.4 eq.) pre-dissolved in deionized water were added via syringe. After copper sulfate’s addition, a yellowish, viscous precipitate was formed. The mixture was stirred for 24 h. Then, ethylenedinitrilotetraacetic acid (0.556 g, 2.286 mmol, and 0.8 eq.) was added, and the mixture was left to stir for additional 2 h until the precipitate was fully dissolved. Subsequently, the solvent was evaporated under reduced pressure. The crude product was purified by reversed-phase chromatography (water:acetonitrile; Puriflash 15C18HP-F0120 from 5 to 100% acetonitrile for 40 min). A total of 2.755 g (1.845 mmol) of white, amorphous powder was received after purification at a yield of 78%. ^1^H NMR (400 MHz, DMSO-*d_6_*) δ 8.75 (s, 1H), 8.81 (d, 1H, J = 4.82), 8.31 (d, 1H, J = 8.39), 7.82–7.74 (m, 2H), 7.39–7.23 (m, 2H), 7.23–7.05 (m, 7H), 7.05–6.95 (m, 2H), 6.68–6.58 (m, 2H), 6.39–6.33 (m, 1H), 5.38–5.32 (m, 1H), 4.57–4.38 (m, 3H), 4.34–4.15 (m, 4H), 4.13–3.87 (m, 3H), 3.22–3.10 (m, 2H), 3.08–2.83 (m, 6H, CH_2_), 2.83–2.71 (m, 1H), 2.70–2.55 (m, 3H), 2.38–2.11 (m, 13H), 2.11–2.04 (m, 1H), 2.01–1.95 (m, 1H), 1.94–1.30 (m, 22H), 1.30–1.06 (m, 6H), 1.06–0.95 (m, 6H, CH_3_+CH_2_). ^13^C NMR (101 MHz, DMSO-*d_6_*) δ 174.54, 174.51, 173.78, 172.87, 172.82, 172.16, 172.10, 171.55, 171.16, 171.05, 157.31, 155.85, 148.98, 145.99, 142.75, 142.70, 141.86, 141.21, 140.80, 139.86, 139.18, 137.98, 134.21, 133.40, 133.06, 132.62, 130.59, 130.24, 129.96, 129.06, 128.06, 127.19, 126.23, 126.07, 125.80, 122.18, 121.90, 114.99, 73.13, 56.93, 55.00, 54.90, 51.66, 49.54, 46.67, 37.72, 36.90, 36.42, 36.29, 35.74, 34.11, 33.14, 31.80, 31.50, 30.90, 30.68, 30.53, 29.94, 29.80, 29.67, 27.55, 27.37, 26.29, 24.73, 24.58, 24.43, 24.30, 20.33, 18.90, and 16.03. FT-IR: 3308.28 (NH), 3079.76 (Ar), 2940.91 (Ar), 2864.74 (Ar), 1718.26 (C(O)NH), 1649.80 (C(O)NH), 1557.24 (C(O)NH), 1540.84 (C(O)NH), 1517.70 (C(O)NH), 1455.03 (C(O)NH). Elemental Analysis Calculated for C_80_H_102_ClN_11_O_15_ × CF_3_COOH (TFA salt monohydrate): C, 60.60; H, 6.51; N, 9.48. Found: C, 60.18; H, 6.20; N, 9.54. HRMS (ESI) *m/z* 746.8701 (calcd 746.8695 for C_80_H_104_ClN_11_O_15_^2+^ [M+2H]^2+^), *m/z* 757.8597 (calcd 757.8605 for C_80_H_103_ClN_11_O_15_Na^2+^ [M+H+Na]^2+^). LC-MS purity: 99.9%. Retention time: 14.0 min.

### 4.5. Cell Lines

22Rv1 and PC-3 cells were received from the MISIS collection of cell lines (less than ten passages from ATCC stock). All cells were cultured in humidified 37 °C incubators with 5% CO_2_. All cell lines were tested for the absence of mycoplasma. 22Rv1, androgen-responsive PSMA-positive human prostate carcinoma cells, were cultured in RPMI 1640 media (Gibco) with 10% FBS (Gibco), 1× GlutaMax (Gibco), and 1× Penicillin-Streptomycin (10,000 U/mL, Gibco). PC-3, PSMA-negative human prostate cancer cells, were cultured in DMEM/F12 media (Gibco) with 10% FBS (Gibco), 1× GlutaMax (Gibco), and 1× Penicillin-Streptomycin (10,000 U/mL, Gibco).

Human adult dermal fibroblasts (HDFa). (ATCC) were propagated in DMEM medium (Paneco) supplemented with 10% fetal calf serum (Gibco) and 1% pemicillin/streptomycin in a 5% CO_2_ atmosphere at 37 °C. Cytotoxicity of **PSMA-Abi** and **AbiAc** in fibroblasts were studied using MTT-test. The cells in the exponential growth phase were trypsinized, counted, and seeded at a density of 5000 cells/well in 96-well plate. Cells were incubated with both tested agents at the designated concentrations (0.4–100 μM) for 72 h; then, each well was treated with 20 μL MTT solution in PBS (5 mg/mL) and cells were incubated at least for 3 h, and formazan was dissolved in DMSO. The absorbance was measured at 450 nm and 620 nm using Thermofischer Scientific Multiscan FC. The values for each point were calculated from 6 wells.

### 4.6. Single Cell ROS Measurement by Using Pt-Nanoelectrodes

Commercially available, disk-shaped carbon nanoelectrodes isolated in quartz (ICAPPIC Limited, London, UK) with diameters of 60–100 nm were used to prepare Pt nanoelectrodes for the amperometric detection of intracellular ROS level. Preparation of Pt-nanoelectrodes has been described in detail previously [43,44].

Briefly, a disk-shaped carbon nanoelectrode was etched in a 0.1 M NaOH, 10 mM KCl solution for 20–40 cycles of 10 seconds (from 0 to +2200 mV) to create nanocavities. Electrochemical deposition of platinum in nanocavities was achieved by cycling from 0 to −800 mV with a scan rate of 200 mV s^−1^ for 8 to 10 cycles in 2 mM H_2_PtCl_6_ solution in 0.1 M hydrochloric acid. Cyclic voltammetry in a 1 mM solution of ferrocene in methanol in PBS was used to control the electrode surface at all stages of fabrication. Prior to the measurements, each platinum electrode was amperometrically calibrated using a series of standard H_2_O_2_ solutions at a potential of +800 mV.

The setup for amperometric measurements included a PC that was connected to a system consisting of an ADC–DAC converter Axon Digidata 1550B (Axon Instruments, Burlingame, CA, USA) and patch-clamp amplifier MultiClamp 700B (Axon Instruments, USA). The working head of the amplifier was fixed on a PatchStar Micromanipulator (Scientifica, London, UK), which was placed near an inverted optical microscope Mikromed-I-LUM (Mikromed, Russia). A Pt nanoelectrode was fixed by a special holder on the working head of the amplifier. Thus, it was possible to change the position of the Pt-nanoelectrode with a micromanipulator and carry out an additional assessment in the optical microscope. The reference electrode was Ag/AgCl. The potential difference between the Pt-nanoelectrode and the reference electrode was recorded with the pClamp 11 software suite (Molecular Devices, Silicon Valley, CA, USA) and processed with Origin 2018 software.

PC-3 (2.0 × 10^5^)/22Rv1 (3.5 × 10^5^) cells were seeded in 35 mm Petri dishes, incubated under the conditions described above, and then treated for 24 h. The complexes (**AbiAc** and **PSMA-Abi** acetate) were dissolved in DMSO, diluted in fresh culture medium with <1% FBS 2 mL, and added to Petri dishes. The final concentration of the compounds in the culture medium was IC_50_ with 1 h of incubation time. Untreated cells were used as a control, which was evaluated at the beginning and at the end of the experiment. Attached cells in Petri dishes were washed three times using Hanks’ Balanced Salt solution to remove the media and traces of complexes. On average, about 10 cells were measured by 2–3 Pt electrodes in 2 independent Petri dishes for each complex in one independent biological replicate. The intracellular ROS level was determined based on the calibration curve. Results are shown as means ± SEM, where n = 3 (three independent biological replicates). Statistical analyses were conducted using one-way ANOVA test in Origin 2021.

### 4.7. Cell Cycle Analysis

Cell cycle was analyzed in 22Rv1 cells fixed in 70% ethanol with Muse^®^ cell cycle kit. The fixation lasted overnight at −20 °C. Then, cells were collected by centrifugation at 4000 rpm for 10 min and washed with PBS. The cells were resuspended with Muse^®^ Cell Cycle reagent and incubated for 30 min at room temperature in the dark; then, they were counted.

### 4.8. Human Tumor Xenograft Mouse Model

Male, 6–8 week old Balb/c nude mice with body weight range of 20–22 g were purchased from N.N.Blokhin Cancer Research Center. Animals were kept in plastic cages equipped with lids with feeding recesses, air filters, and feed dividers at 2–3 mice per cage.

Mice were injected subcutaneously in the right and left flanks with 1 × 10^7^ 22Rv1 cells in RPMI-1640 medium 0.2 mL supplemented with 0.2 mL Corning Matrigel^®^ matrix (Corning, NY, USA) in a total volume of 400 μL. When the average size of the tumors reached ~100 mm^3^, mice were randomly divided into three groups, with five mice per group, including a vehicle group (1% starch paste) and two treatment groups: **PSMA-Abi** 500 mg/kg/day and **AbiAc** 200 mg/kg/day for 14 days. The drugs were administered orally, and all compounds were formulated with 1% starch paste. Following the treatment, the primary tumor size was measured twice weekly with a micro-caliper, and the tumor volume was calculated using the formula: Volume = π/6 × length × width × height. Treatment outcome was based on percentage of tumor growth inhibition (%TGI), defined as the percent difference between the median tumor volumes (MTVs) of mice in the treated and control groups. Percent TGI was calculated using the following formula: %TGI = [1 − (MTV treatment group/MTV control)] 100%. All data are presented as means ± SD. Statistical analyses of in vivo research were performed using SPSS Statistics V. 22.0, comparing continuous variables by non-parametrical Mann–Whitney U. * *p* ≤ 0.05 was considered as significant in comparison with the control.

### 4.9. Acute Toxicity

White, outbred male mice (ICR) about 2 months old weighing 19–21 g were used. Conjugate **PSMA-Abi** (2000 mg/kg) was formulated with 1% starch gel solution. The mixture was administrated intragastrically in an amount not exceeding 0.5 mL, and in portions if necessary. The control group was administered 1% starch gel solution. Animals were kept under controlled environmental conditions: air temperature 20–26 °C and relative humidity 30–70%. Lighting in the rooms was artificial (a cycle of 12 h of light, 12 h of darkness). Before the start of the study, animals were divided into groups of 10 animals per group. The distribution was made such that the average masses of groups of animals of one species differed by no more than 10%. Before the experiment concerning the determination of acute toxicity, the animals were deprived of food and water (mice for 2 h) and then weighed. The duration of observation of laboratory animals was 14 days. During this period, visible signs of intoxication were evaluated. Dose selection was carried out based on 22Rv1 xenograft experiments with the maximum effective dose for mice of 500 mg/kg.

### 4.10. Pharmacokinetics

The pharmacokinetic parameters were studied for healthy, outbred, adult, male, albino rats (Wistar) about 3 months old weighing 190–210 g. The experiment included 3 rats for preparation. The formulation of conjugate **PSMA-Abi** was the same as for the acute toxicity evaluation. Administration dose was 1000 mg/kg.

Blood samples were taken from the tail vein of rats in a volume of 0.2 mL in polyethylene micro-tubes with a capillary up to 0.5 mL by trimming the tip of the tail. K2EDTA was used as an anticoagulant. During the study, a total of 48 blood samples from 3 animals (16 samples from each animal) were selected for **PSMA-Abi**.

Immediately after taking a blood sample, the tube was carefully inverted several times to mix the contents (anticoagulant). The plasma was then separated by centrifugation at 1500× *g* for 10 min at a temperature of +4 °C. Centrifugation was performed no later than 15 min after sampling each blood sample. The studied conjugate **PSMA-Abi** was determined in the blood plasma samples of rats and rabbits by high-performance liquid chromatography (Agilent 1200) with tandem mass spectrometric detection (QTrap 4500 tandem mass spectrometer) with preliminary isolation of the analyte from the biomaterial. Detailed method parameters are described in Table S6.

### 4.11. Study of PSMA-Abi’s Ability to Inhibit the Activity of Human Liver Cytochromes (1A2, 2C8, 2C9, 2C19, 2D6, and 3A4)

To assess the effect of drugs on liver cytochrome activity in drug development, test systems with recombinant enzymes and fluorescent substrates as well as microsomes with metabolic substrates and HPLC-MS/MS analysis were used. The present study aimed to investigate the effect of targeted delivery drugs specific to malignant prostate cells on cytochrome P450 (CYP450) isoenzyme activity in human liver microsomes using conventional sample substrates and HPLC-MS/MS detection. Detailed methodological parameters are described in the Supplementary Material.

## Figures and Tables

**Table 1:** Cytotoxicity of conjugate **PSMA-Abi** in comparison with **AbiAc** on 22Rv1 PC-3 and HDFa cell lines.

Compound	CC_50_, μM		
	PC-3	22Rv1	HDFa
**PSMA-Abi**	8.1 ± 2.8	8.2 ± 3.1	>100
**AbiAc**	5.9 ± 0.8 [41]	22.7 ± 3.3	>100

**Table 2:** Pharmacokinetic parameters of conjugate **PSMA-Abi** estimated in rats after single peroral administration at a dose 1000 mg/kg as 1% starch suspension formulation.

	Mean ± SD
C_max_, ng/mL	318 ± 190
**T_max_**, h	3.8
**AUC_0–t_**, h× ng/mL	3791 ± 875
**AUC_0–∞_**, h× ng/mL	4623 ± 1260
**AUC_t–∞_/AUC_0–∞_**, %	21 ± 5
**K_el_**, 1/h	0.023 ± 0.006
**T_1/2_**, h	31.3 ± 8.7
**MRT_0–t_**, h	27.2 ± 1.7

**Table 3:** Inhibition of series of P450 cytochromes (1A2, 3A4-M, 3A4-T, 2C8, 2C9, 2C19, and 2D6) by **PSMA-Abi** and a series of reference substances.

Cytochrome	Substrate	Standard Inhibitor	IC_50,_ nM	PSMA-Abi, µM
1A2	Phenacetin	α-Naphthoflavone	57 ± 6	4.5 ± 1.1
3A4-M	Midazolam	Ketoconazole	90 ± 8	1.1 ± 0.2
3A4-T	Testosterone	Ketoconazole	184 ± 41	5.7 ± 0.8
2C8	Paclitaxel	Montelukast	1270 ± 262	4.4 ± 1.1
2C9	Tolbutamide	Sulfaphenazole	641 ± 77	>25
2C19	Mephenytoin	Tranylcypromine	11,700 ± 940	>25
2D6	Dextromethorphan	Quinidine	64 ± 5	17 ± 1

## Data Availability

Data is given in the manuscript and Supplementary Materials.

## Supplementary Materials

The following supporting information can be downloaded at: https://www.mdpi.com/article/10.3390/molecules27248795/s1, Figure S1: Pharmacokinetic curves for each rat (in linear and semi-logarithmic coordinates); Figure S2: Average rat pharmacokinetic profiles (in linear and semilogarithmic coordinates); Figure S3: Cytotoxicity curves for PSMA-Abi on 22Rv1 (A) and PC-3 cells (B); Abi on 22Rv1 cells (C); PSMA-Abi and AbiAc on fibroblasts (D); Figure S4: Cell cycle distribution obtained on 22Rv1 cells after incubation with PSMA-Abi and AbiAc; Table S1: Tumor growth inhibition on xenografts tumor models; Table S2: Tumor volume and statistical analysis on xenografts tumor models; Table S3: Weight dynamic of mice males in single injection of conjugate PSMA-Abi experiments in comparison with control group, % of starting weight; Table S4: Concentrations of conjugate 6 in the blood plasma of rats and statistics description for them; Table S5: Pharmacokinetic parameters obtained on rats; Table S6: Chromatography parameters in pharmacokinetic evaluation; Table S7: Preparation of microsomes solution; Table S8: Preparation of the Starting solution of substrates; Table S9: Preparation of a combined solution of substrates; Table S10: Preparation of starting solutions of metabolites; Table S11: Preparation of combined solutions of metabolites; Table S12: Preparation of the NANPR regeneration system.

## Sample Availability

Samples of the compounds are not available from the authors.

## Abbreviations

2-PMPA: 2-Phosphonomethyl pentanedioic acid; CYP, Cytochrome P450; CRPC, Castration-resistant prostate cancer; DIPEA, N, N-Diisopropylethylamine; DMF, N, N-Dimethylformamide; DMSO, dimethyl sulfoxide; FBS, fetal bovine serum; FDA, food and drug administration; K2EDTA, ethylenedinitrilo) tetraacetic acid dipotassium salt; MMAE, monomethyl auristatin E; PSA, prostate-specific antigen; PSMA, prostate-specific membrane antigen; ROS, reactive oxygen species; SICM, scanning ion conductance microscopy; TGI, tumor growth inhibition; TLC, thin-layer chromatography.
