# Supplementary material for: Synthesis and Preclinical Evaluation of Small-Molecule Prostate-Specific Membrane Antigen-Targeted Abiraterone Conjugate
Source: Molecules. 2022 Dec 12;27(24):8795. doi: 10.3390/molecules27248795 (PMC9783881; doi:10.3390/molecules27248795)
Supplement: Supplementary file 1 [file molecules-27-08795-s001.zip › molecules-2038124-supplementary.pdf]

Synthesis of Abiraterone (2).

¹H NMR

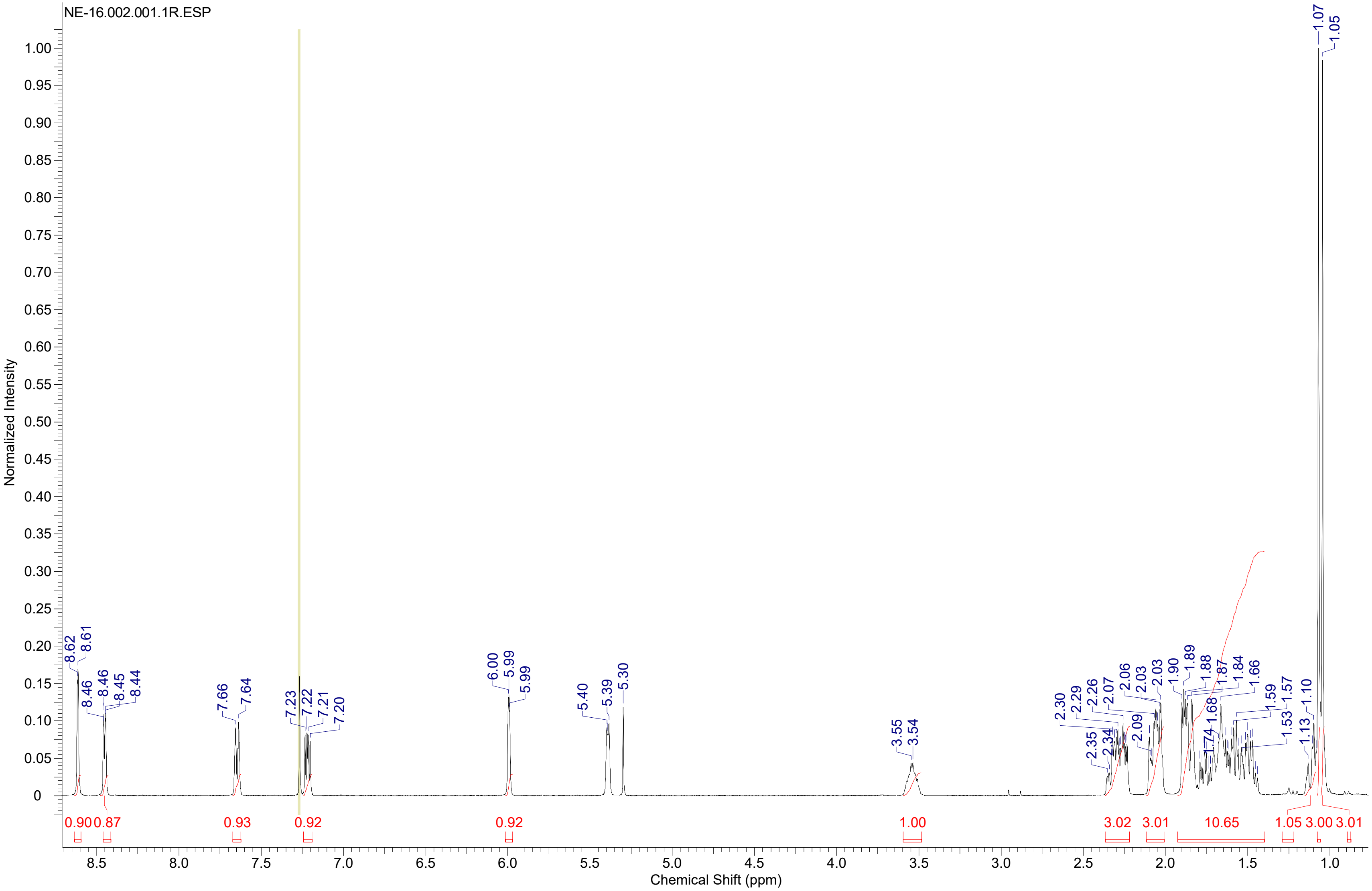

^{13}C NMR

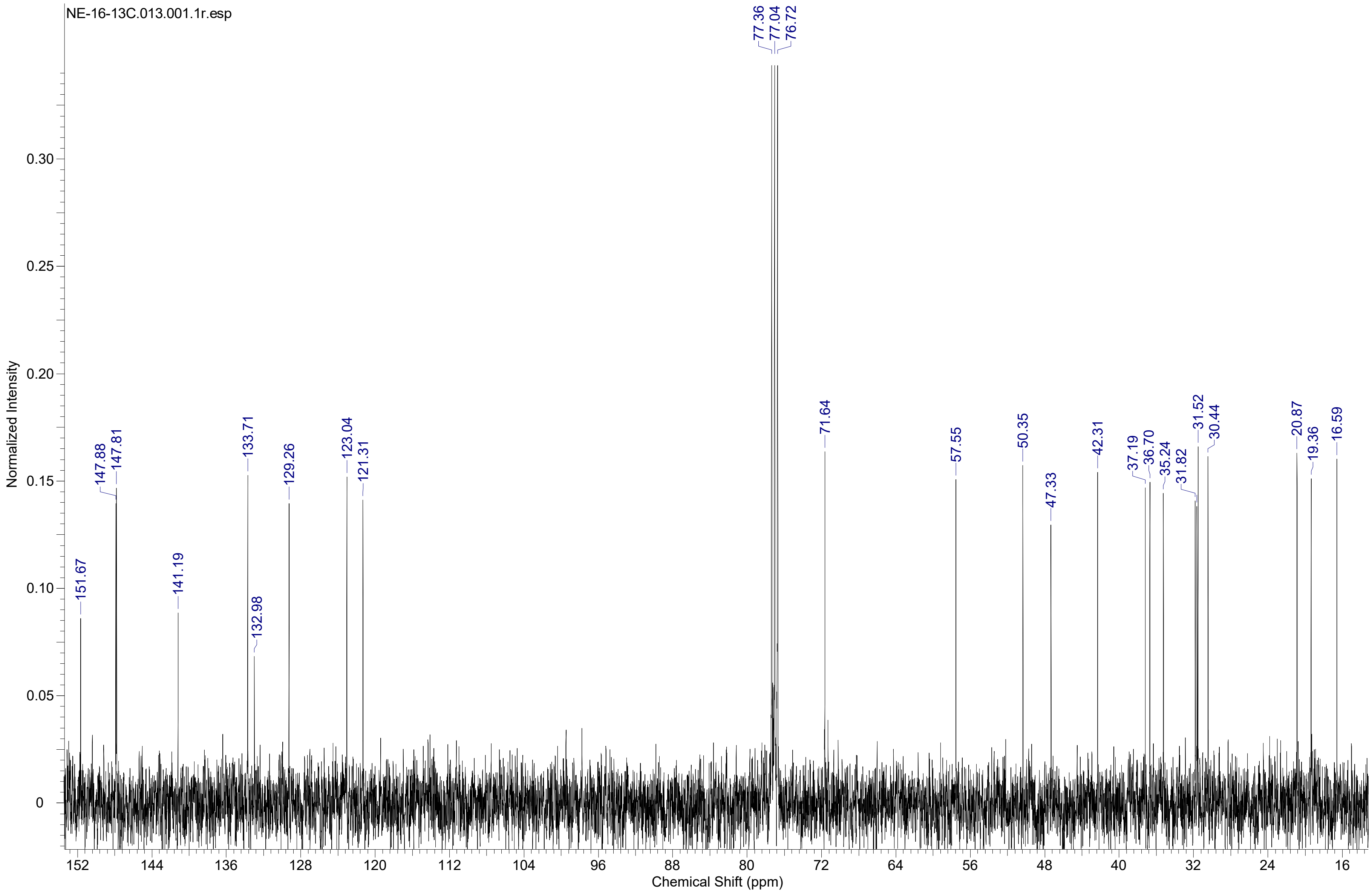
NE-16-13C.013.001.1r.esp
77.36
77.04
76.72
151.67
147.88
147.81
141.19
133.71
132.98
129.26
123.04
121.31
71.64
57.55
50.35
47.33
42.31
37.19
36.70
35.24
31.82
31.52
30.44
20.87
19.36
16.59
Normalized Intensity
Chemical Shift (ppm)

Abiraterone hexyn-5-oate (3).

^{1}H NMR

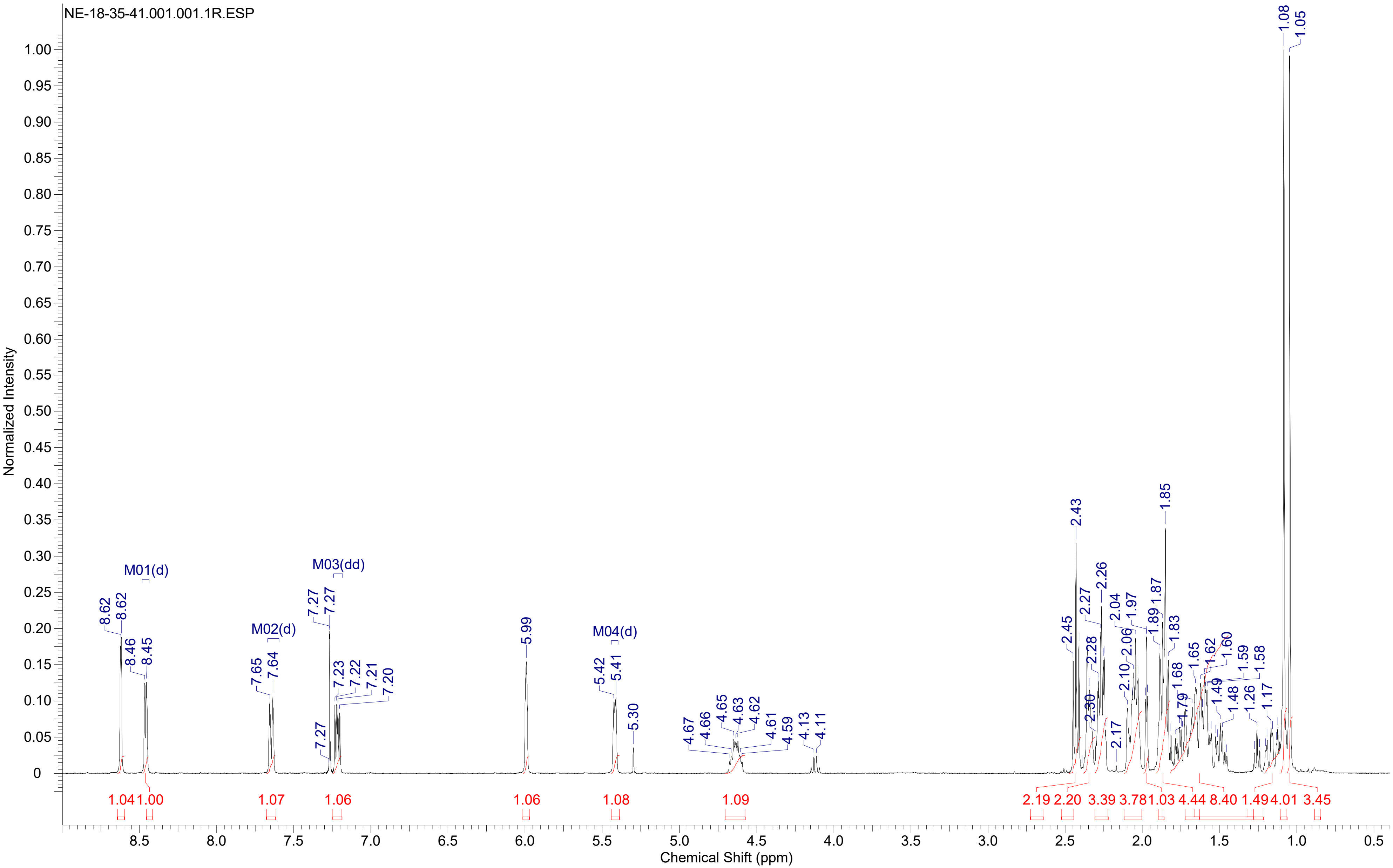

^{13}C NMR

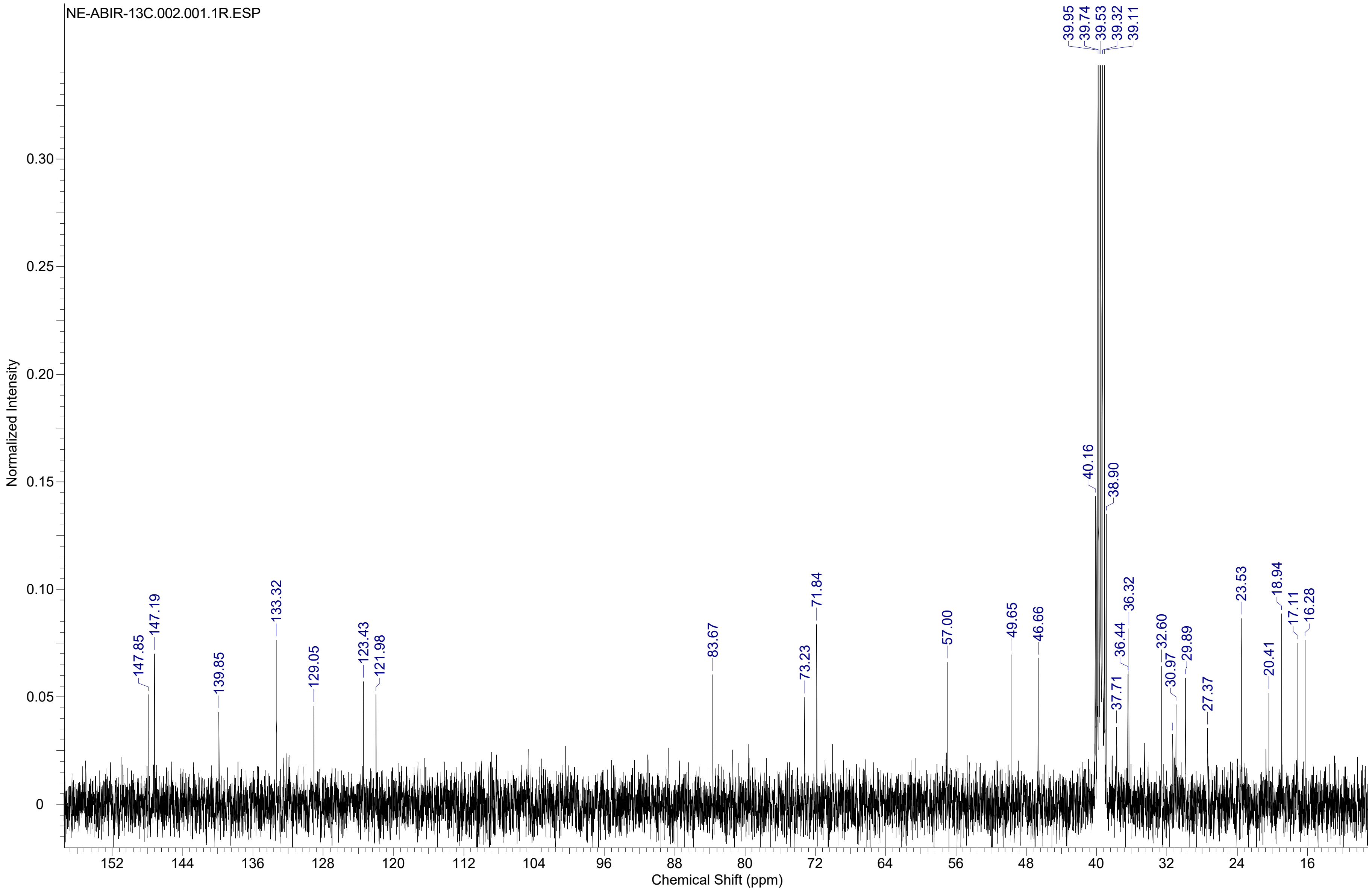

NE-ABIR-13C.002.001.1R.ESP
39.95
39.74
39.53
39.32
39.11
40.16
38.90
147.85
147.19
139.85
133.32
129.05
123.43
121.98
83.67
73.23
71.84
57.00
49.65
46.66
37.71
36.44
36.32
32.60
30.97
29.89
27.37
23.53
20.41
18.94
17.11
16.28
Normalized Intensity
0.30
0.25
0.20
0.15
0.10
0.05
0
152
144
136
128
120
112
104
96
88
80
72
64
56
48
40
32
24
16
Chemical Shift (ppm)

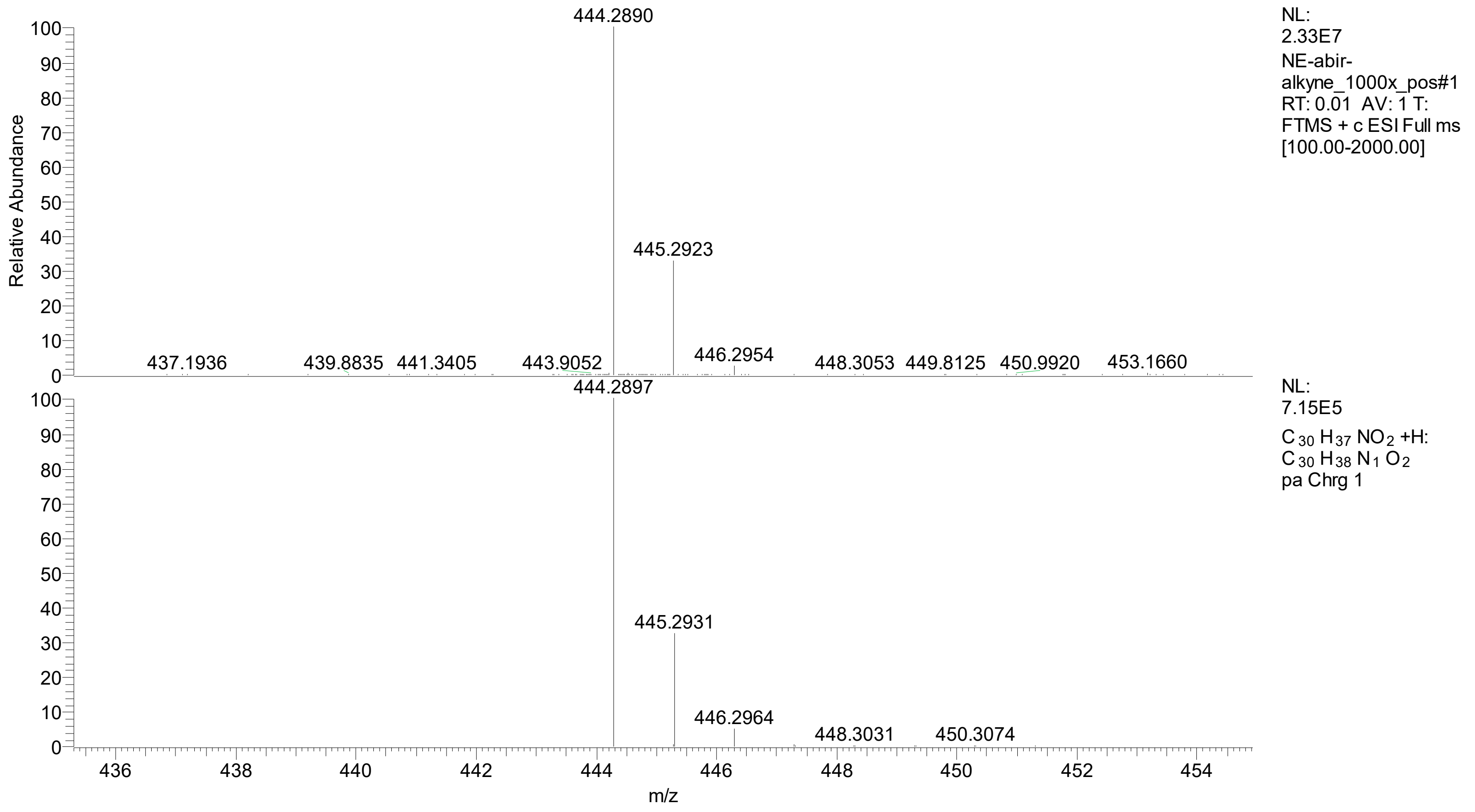

NL:
2.33E7
NE-abir-
alkyne_1000x_pos#1
RT: 0.01 AV: 1 T:
FTMS + c ESI Full ms
[100.00-2000.00]
NL:
7.15E5
C 30 H 37 NO 2 +H:
C 30 H 38 N 1 O 2
pa Chrg 1
Relative Abundance
m/z
444.2890
445.2923
446.2954
437.1936
439.8835
441.3405
443.9052
448.3053
449.8125
450.9920
453.1660
444.2897
445.2931
446.2964
448.3031
450.3074

PSMA-Abiraterone (4)

^{1}H NMR

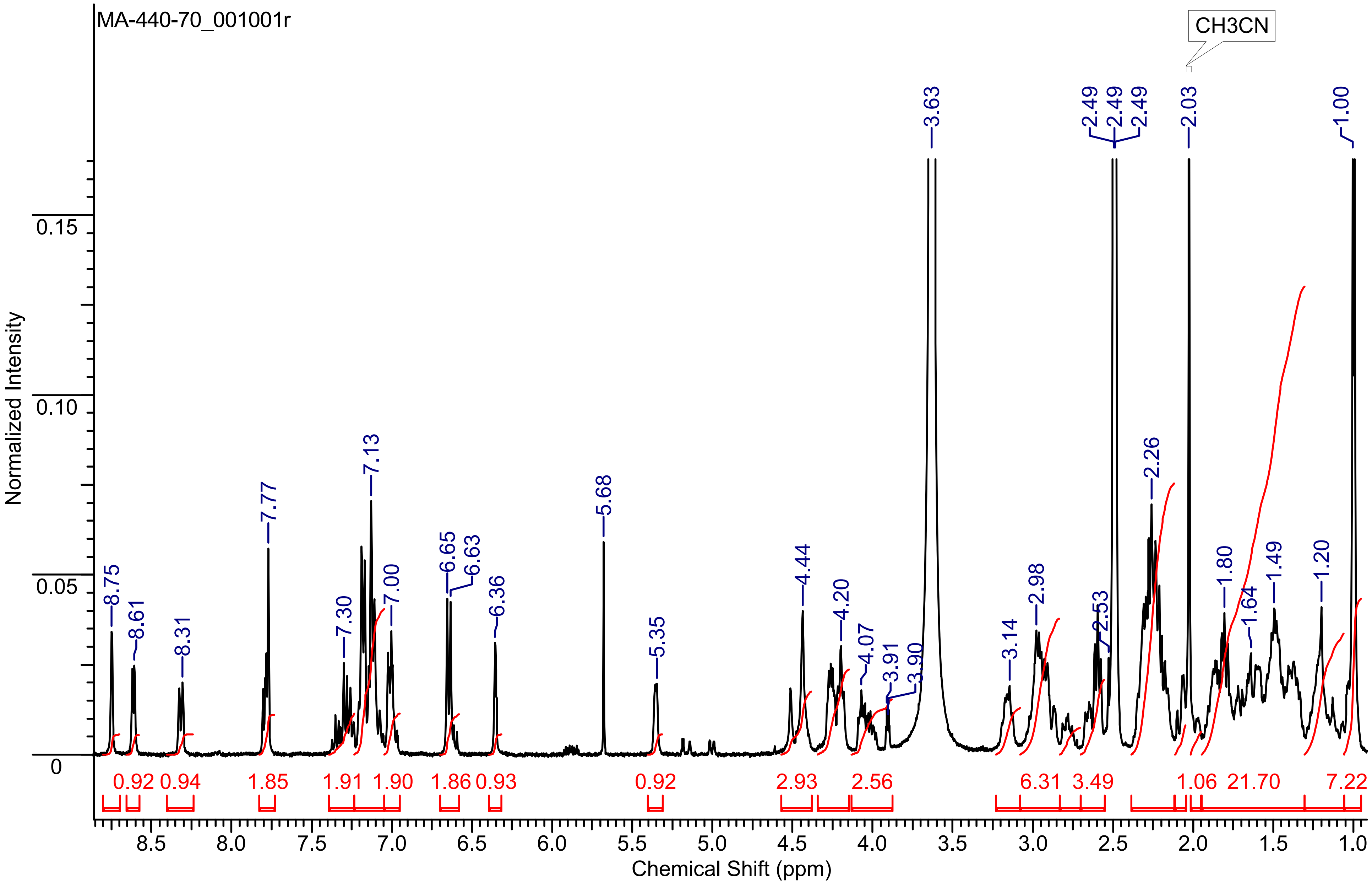

^{13}C NMR

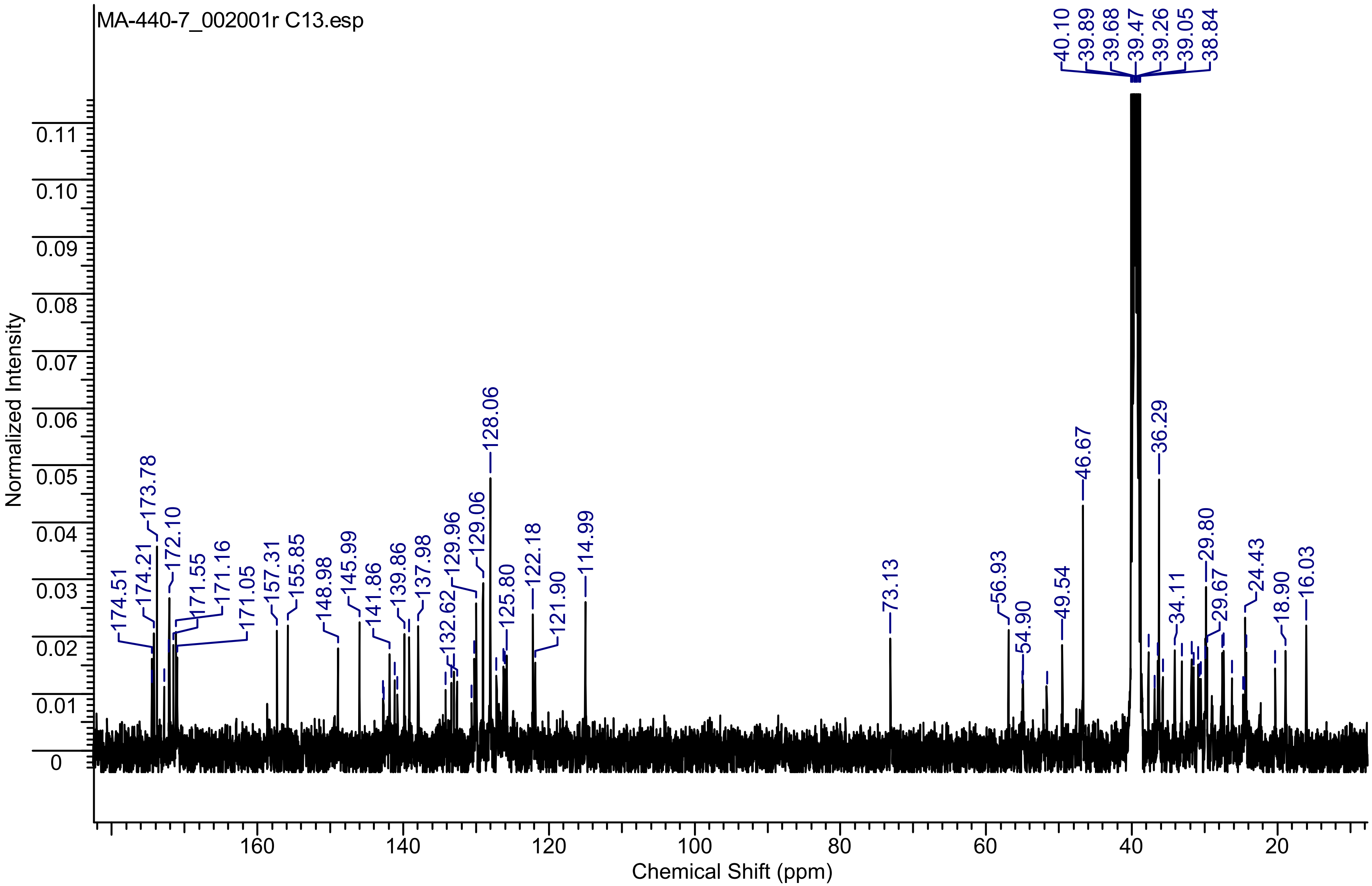

MA-440-7_002001r C13.esp
Normalized Intensity
Chemical Shift (ppm)
174.51
174.21
173.78
172.10
171.55
171.16
171.05
157.31
155.85
148.98
145.99
141.86
139.86
137.98
132.62
129.96
129.06
128.06
125.80
122.18
121.90
114.99
73.13
56.93
54.90
49.54
46.67
40.10
39.89
39.68
39.47
39.26
39.05
38.84
36.29
34.11
29.80
29.67
24.43
18.90
16.03

FT-IR

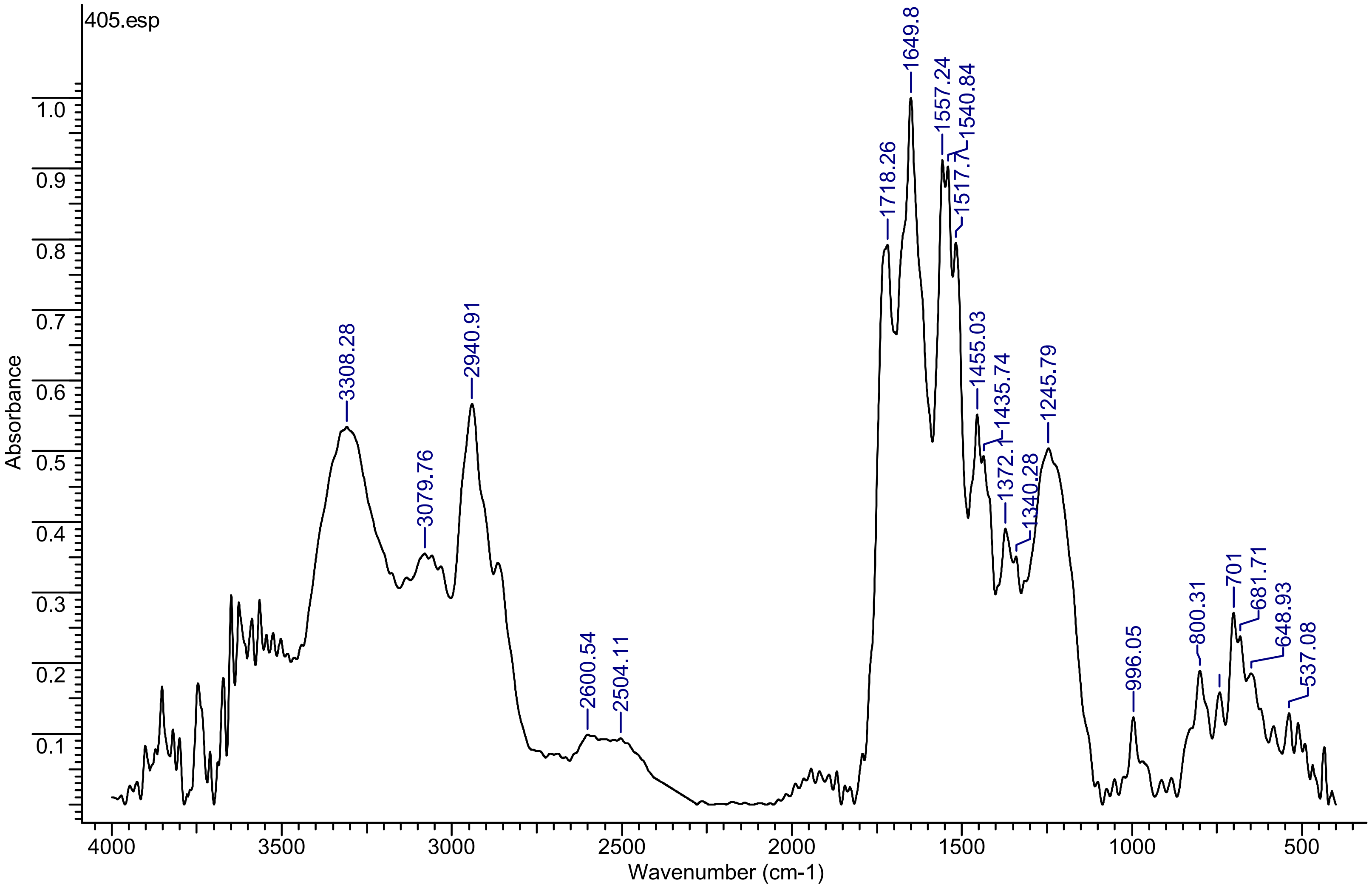

405.esp
Absorbance
Wavenumber (cm-1)
3308.28
3079.76
2940.91
2600.54
2504.11
1718.26
1649.8
1557.24
1540.84
1517.7
1455.03
1435.74
1372.1
1340.28
1245.79
996.05
800.31
701
681.71
648.93
537.08

LCMS

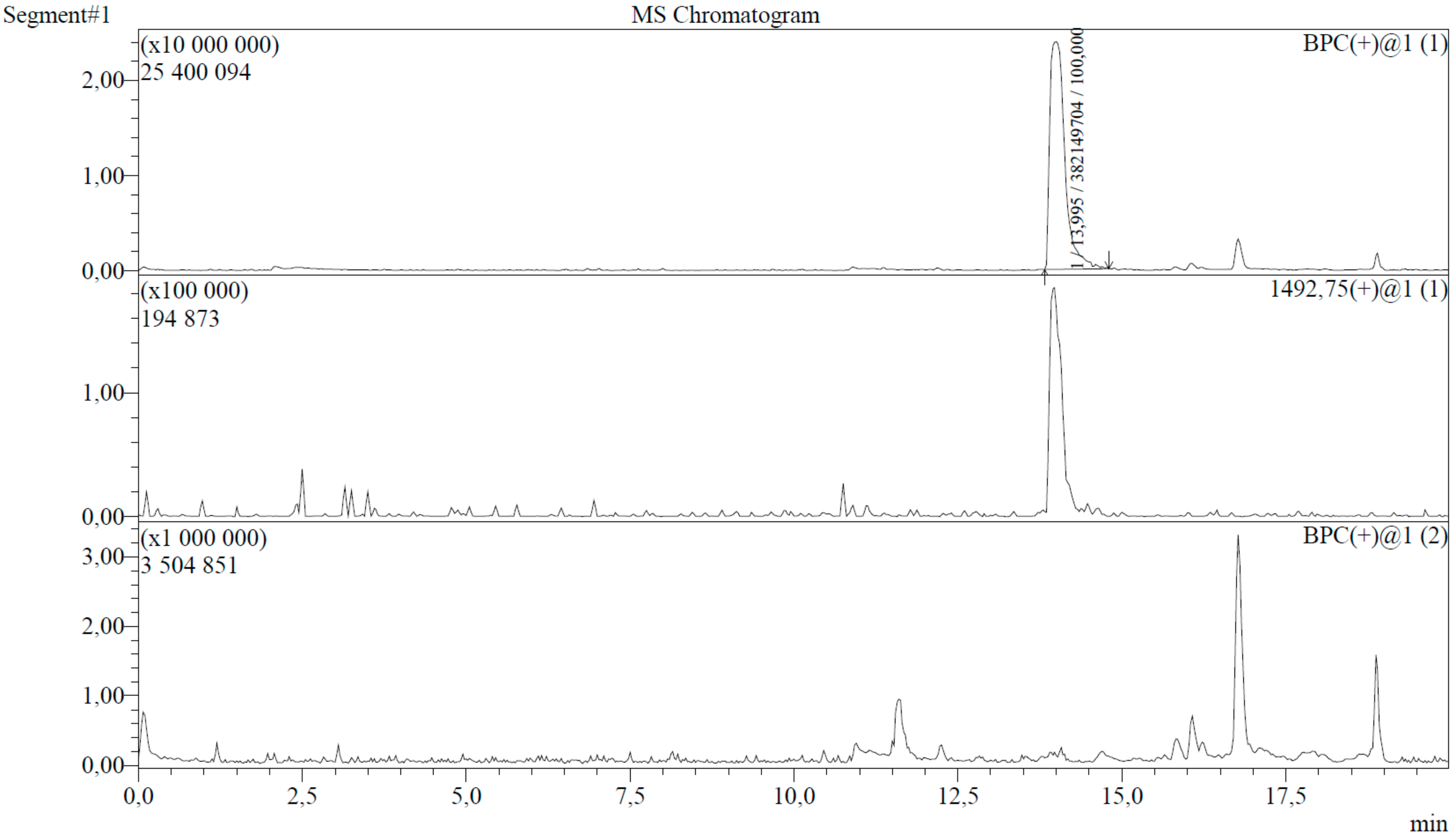
Segment#1
MS Chromatogram
(x10 000 000)
25 400 094
BPC(+)@1 (1)
1 / 13,995 / 382149704 / 100,000
2,00
1,00
0,00
(x100 000)
194 873
1492,75(+)@1 (1)
1,00
0,00
(x1 000 000)
3 504 851
BPC(+)@1 (2)
3,00
2,00
1,00
0,00
0,0
2,5
5,0
7,5
10,0
12,5
15,0
17,5
min

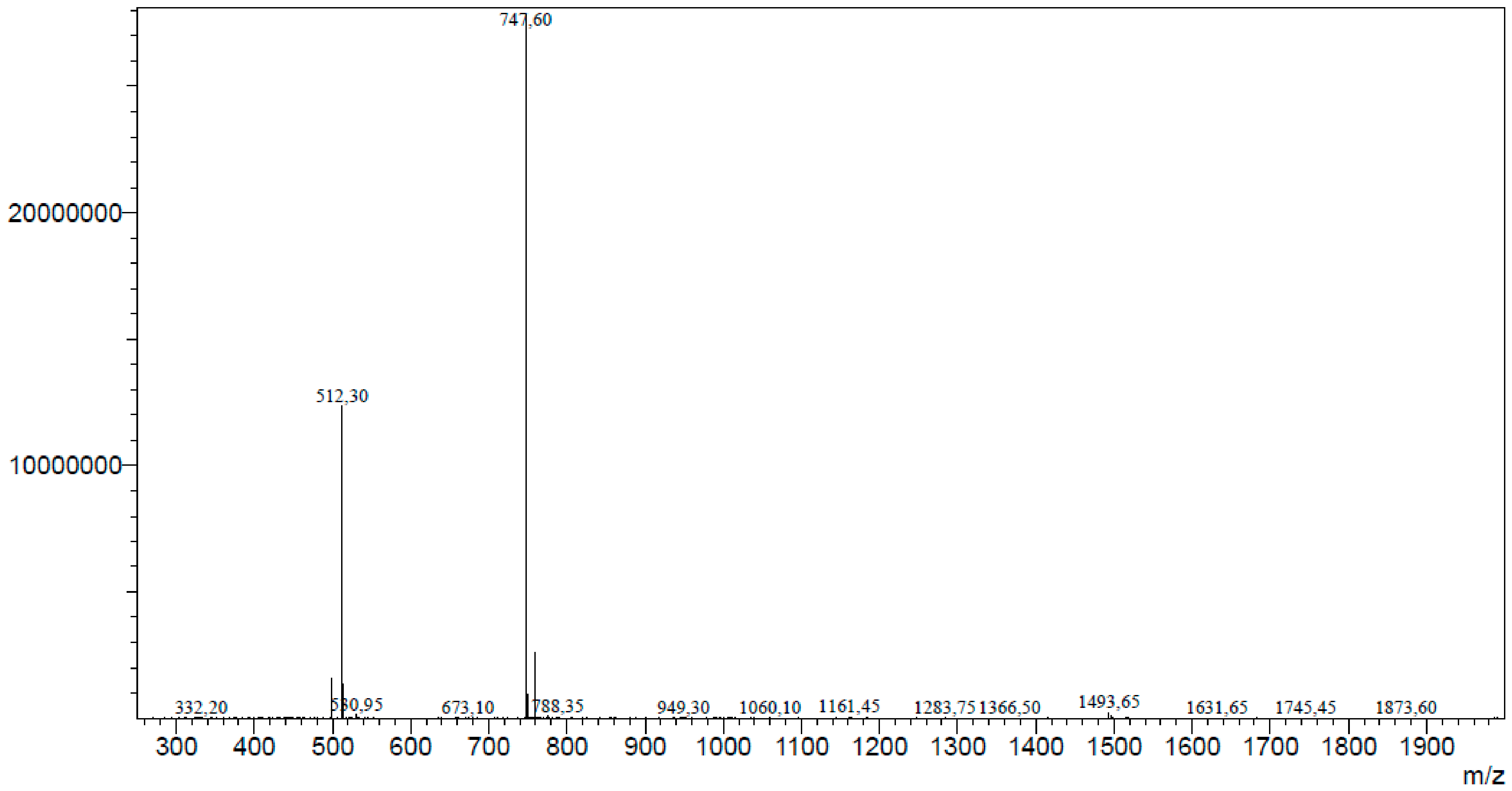
747,60
512,30
20000000
10000000
332,20
530,95
673,10
788,35
949,30
1060,10
1161,45
1283,75
1366,50
1493,65
1631,65
1745,45
1873,60
300
400
500
600
700
800
900
1000
1100
1200
1300
1400
1500
1600
1700
1800
1900
m/z

HRMS (ESI)

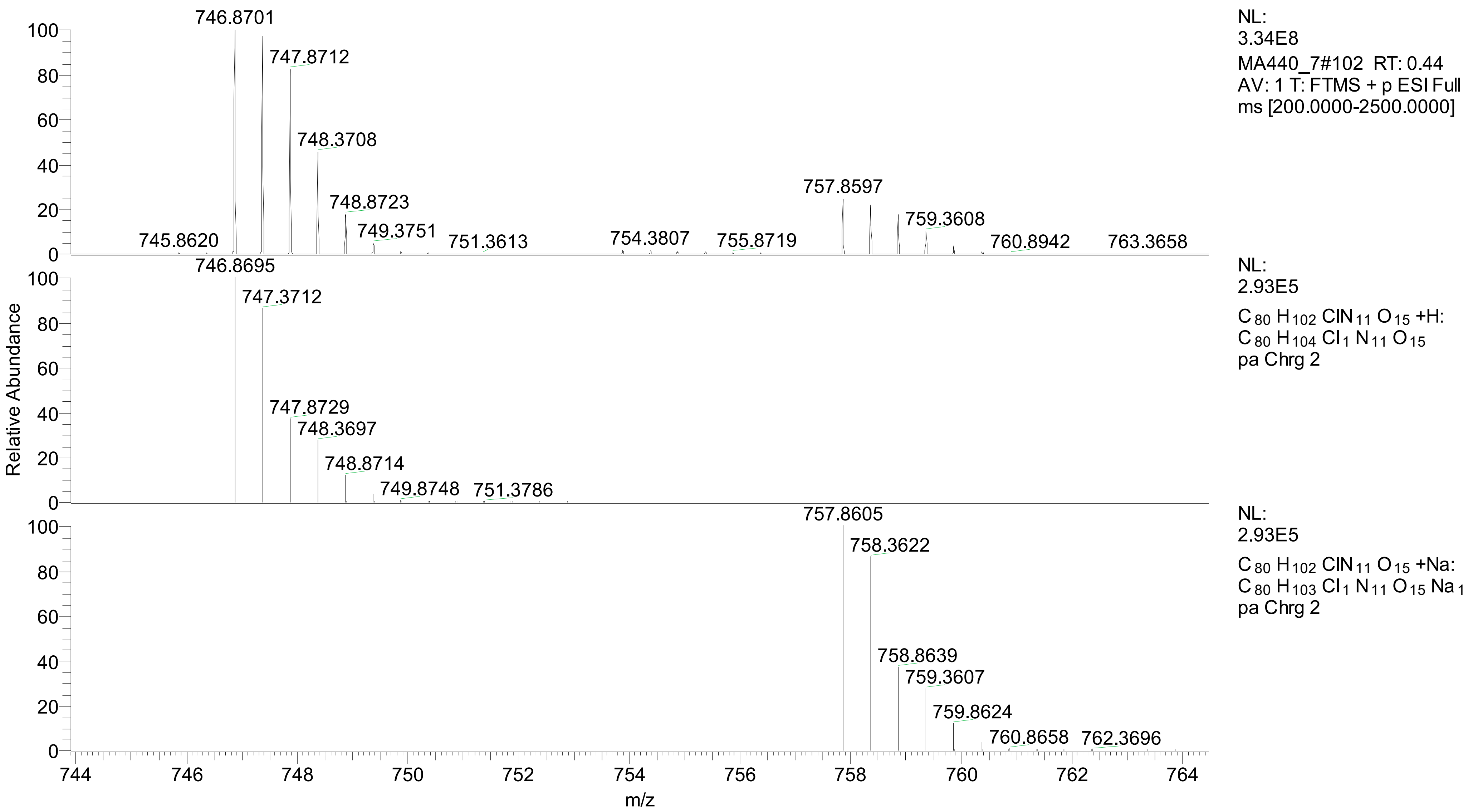

746.8701
747.8712
748.3708
748.8723
749.3751
745.8620
751.3613
754.3807
755.8719
757.8597
759.3608
760.8942
763.3658
NL:
3.34E8
MA440_7#102 RT: 0.44
AV: 1 T: FTMS + p ESI Full
ms [200.0000-2500.0000]
746.8695
747.3712
747.8729
748.3697
748.8714
749.8748
751.3786
NL:
2.93E5
C 80 H 102 ClN 11 O 15 +H:
C 80 H 104 Cl 1 N 11 O 15
pa Chrg 2
757.8605
758.3622
758.8639
759.3607
759.8624
760.8658
762.3696
NL:
2.93E5
C 80 H 102 ClN 11 O 15 +Na:
C 80 H 103 Cl 1 N 11 O 15 Na 1
pa Chrg 2
Relative Abundance
m/z

Efficacy evaluation

Table S1. Tumor growth inhibition on xenografts tumor models

Day after start of the treatment	**15**	**18**	**22**
	TGI, %		
**PSMA-Abiraterone**	65	63	60
**Abiraterone acetate**	78	72	69

Table S2. Tumor volume and statistical analysis on xenografts tumor models

Day after start of the treatment	**0**	**15**	**18**	**22**
Tumor volume, mm^3 (M+SE)				
**Control**	145±22	2480±244	2867±255	3260±263
**PSMA-Abiraterone**	110±18	876±197	1063±195	1294±201
p (PSMA-Abiraterone/ Control)	-	p<0,001*	p<0,001*	p<0,001*
**Abiraterone acetate**	91±20	547±64	811±72	997±110
p (PSMA-Abiraterone/ Control)	-	p<0,001*	p<0,001*	p<0,001*
p (PSMA-Abiraterone/ Abiraterone acetate)	-	0,210	0,218	0,353

Acute toxicity

Table S3. Weight dynamic of mice males in single injection of conjugate **PSMA-Abi** experiments in comparison with control group, % of starting weight.

Group	Dose, mg/kg	Mouse №	5 days	10 days	14 days
1	0	1	20.7	41.9	61.1
1	0	2	16.7	33.3	49
1	0	3	26.9	30.8	44.3
1	0	4	26.5	43.4	64.8
1	0	5	23.9	32	54.8
1	0	6	26.3	47	52.5
1	0	7	21.5	30	54
1	0	8	28.6	43.9	66.8
1	0	9	30.5	34.5	52.2
1	0	10	22.9	31.7	59.5
**M±SD**			**24.5±4.1**	**36.9±6.4**	**55.9±7.1**
2	2000	1	31.5	44.8	51.2
2	2000	2	24.3	35.2	35.7
2	2000	3	16.2	35.2	37.6
2	2000	4	4.9	5.9	-14.6
12	2000	5	20.2	34.1	33.2
12	2000	6	15.4	35.6	41.8
2	2000	7	21.5	39.5	41.5
2	2000	8	10.9	17.8	21.3
2	2000	9	17.5	35.0	31.1
2	2000	10	12.7	25.4	27.8
**M±SD**			**17.5±7.4**	**30.9±11.5**	**30.7±17.9**

Pharmacokinetics

Table S4. Concentrations of conjugate **6** in the blood plasma of rats and statistics description for them

	Time after injection, h														
	**0.00**	**0.25**	**0.50**	**1.00**	**1.50**	**2.00**	**3.00**	**4.00**	**6.00**	**8.00**	**10.00**	**12.00**	**24.00**	**48.00**	**72.00**
**Rat**	**Concentration (μg/ml)**														
**1**	0.00	54.09	43.11	80.92	105.54	227.00	36.31	95.95	BLQ	72.20	151.05	162.20	63.28	BLQ	27.11
**2**	0.00	44.78	13.88	11.06	190.03	20.63	39.29	39.62	64.30	58.57	31.47	69.92	BLQ	BLQ	16.76
**3**	0.00	37.91	12.07	112.23	33.63	29.48	33.96	14.63	93.32	536.71	29.45	85.29	BLQ	BLQ	19.47
**N**	**3**	**3**	**3**	**3**	**3**	**3**	**3**	**3**	**2**	**3**	**3**	**3**	**1**	**0**	**3**
**Mean**	**0.00**	**45.59**	**23.02**	**68.07**	**109.73**	**92.37**	**36.52**	**50.07**	**78.81**	**222.49**	**70.66**	**105.80**	**63.28**		**21.11**
**SD**	**0.00**	**8.12**	**17.42**	**51.79**	**78.28**	**116.68**	**2.67**	**41.65**	**20.52**	**272.20**	**69.63**	**49.44**			**5.37**
**Min**	**0.00**	**37.91**	**12.07**	**11.06**	**33.63**	**20.63**	**33.96**	**14.63**	**64.30**	**58.57**	**29.45**	**69.92**	**63.28**		**16.76**
**Median**	**0.00**	**44.78**	**13.88**	**80.92**	**105.54**	**29.48**	**36.31**	**39.62**	**78.81**	**72.20**	**31.47**	**85.29**	**63.28**		**19.47**
**Max**	**0.00**	**54.09**	**43.11**	**112.23**	**190.03**	**227.00**	**39.29**	**95.95**	**93.32**	**536.71**	**151.05**	**162.20**	**63.28**		**27.11**
**Range**	**0.00**	**16.18**	**31.04**	**101.17**	**156.40**	**206.37**	**5.33**	**81.32**	**29.02**	**478.14**	**121.60**	**92.28**	**0.00**		**10.35**
**Geometric mean**		**45.11**	**19.33**	**46.48**	**87.70**	**51.68**	**36.46**	**38.17**	**77.46**	**131.42**	**51.92**	**98.90**	**63.28**		**20.68**

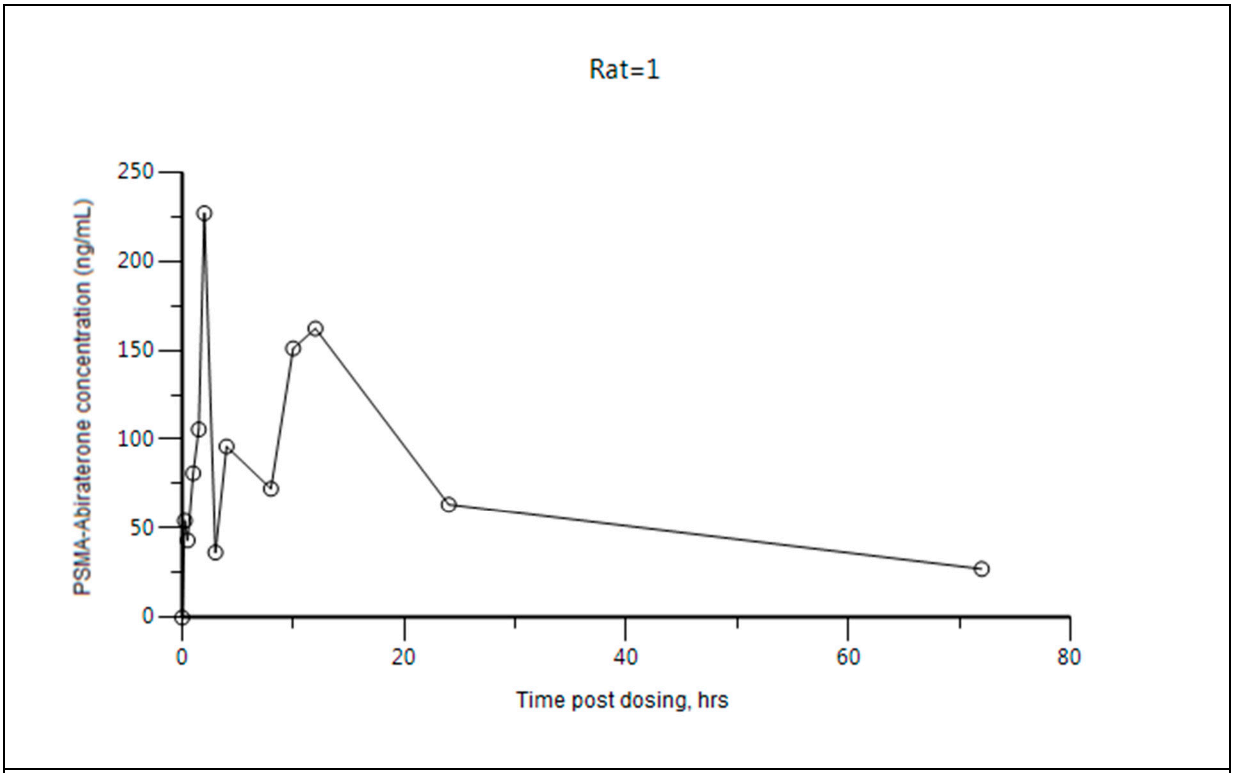
Rat=1
250
200
150
100
50
0
PSMA-Abiraterone concentration (ng/mL)
0
20
40
60
80
Time post dosing, hrs

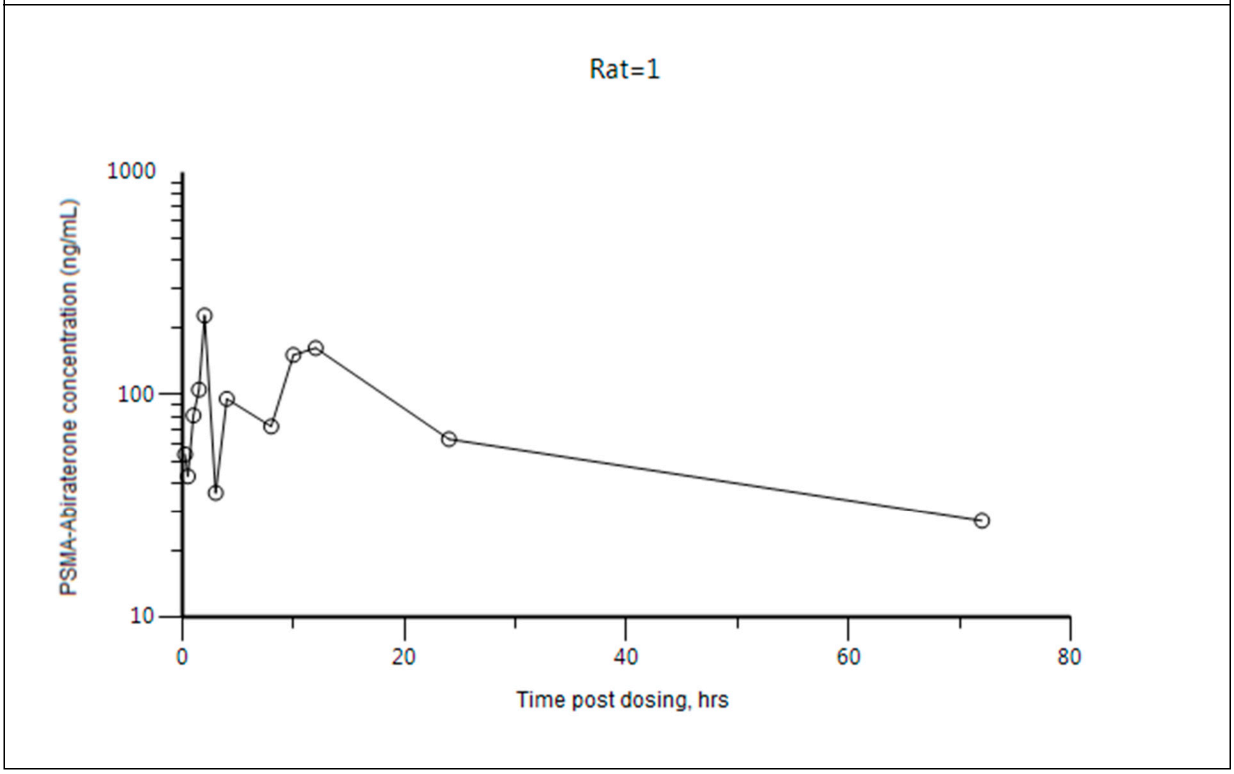
Rat=1
1000
100
10
PSMA-Abiraterone concentration (ng/mL)
0
20
40
60
80
Time post dosing, hrs

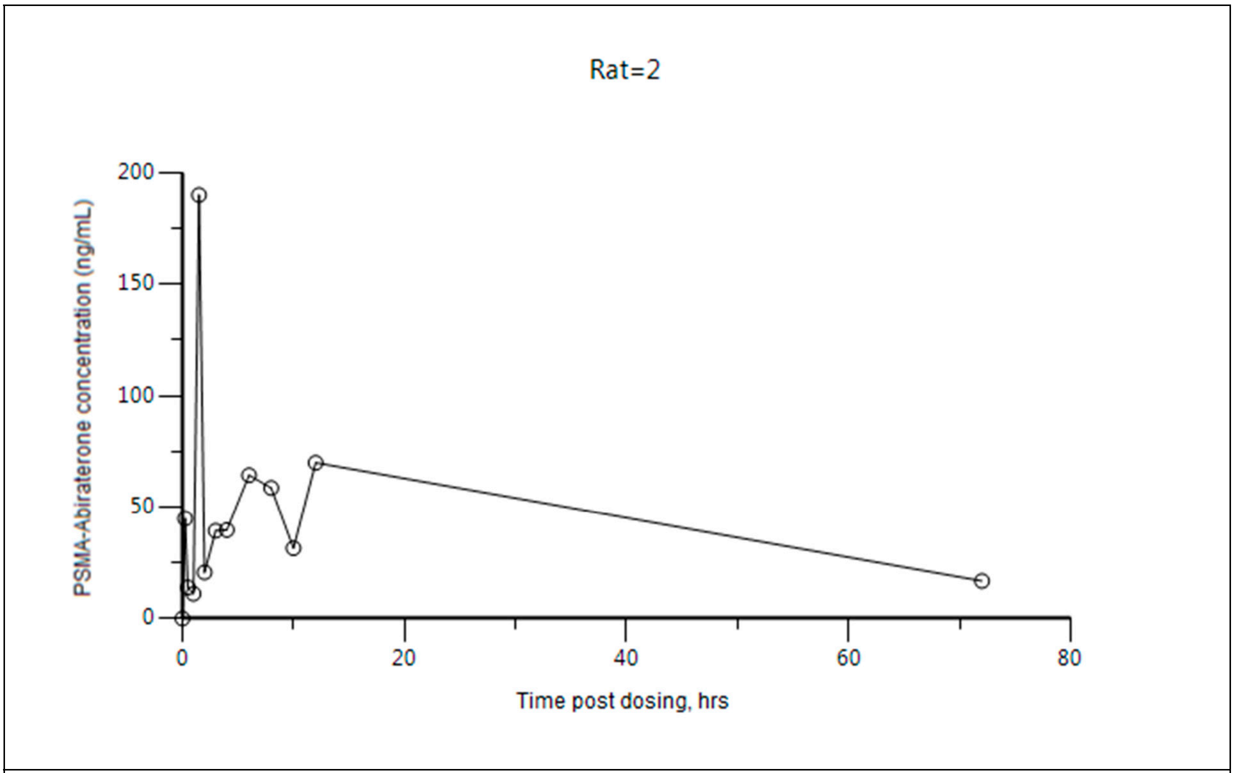

Rat=2
PSMA-Abiraterone concentration (ng/mL)
200
150
100
50
0
0
20
40
60
80
Time post dosing, hrs

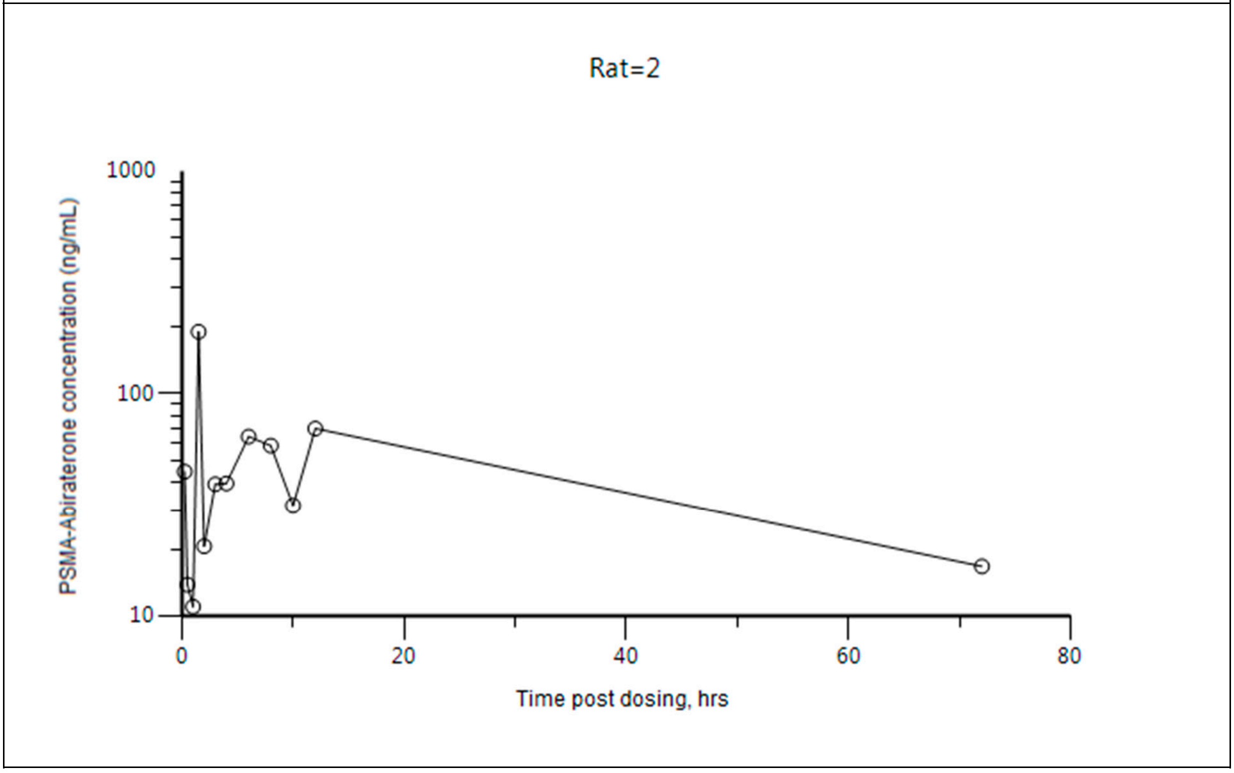

Rat=2
PSMA-Abiraterone concentration (ng/mL)
1000
100
10
0
20
40
60
80
Time post dosing, hrs

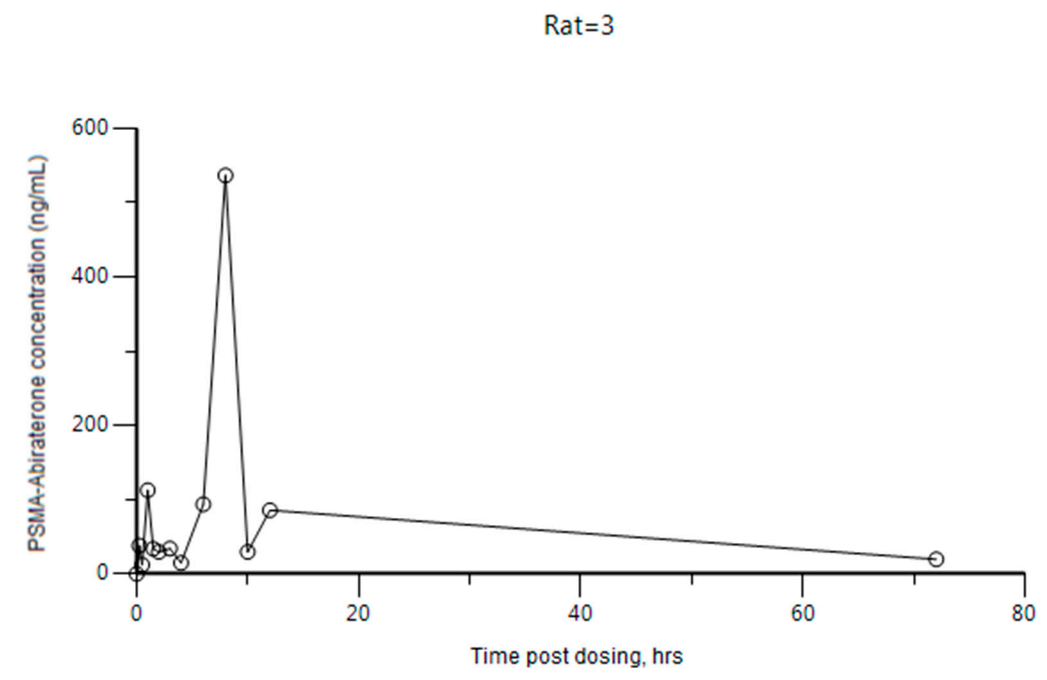
Rat=3
PSMA-Abiraterone concentration (ng/mL)
600
400
200
0
0
20
40
60
80
Time post dosing, hrs

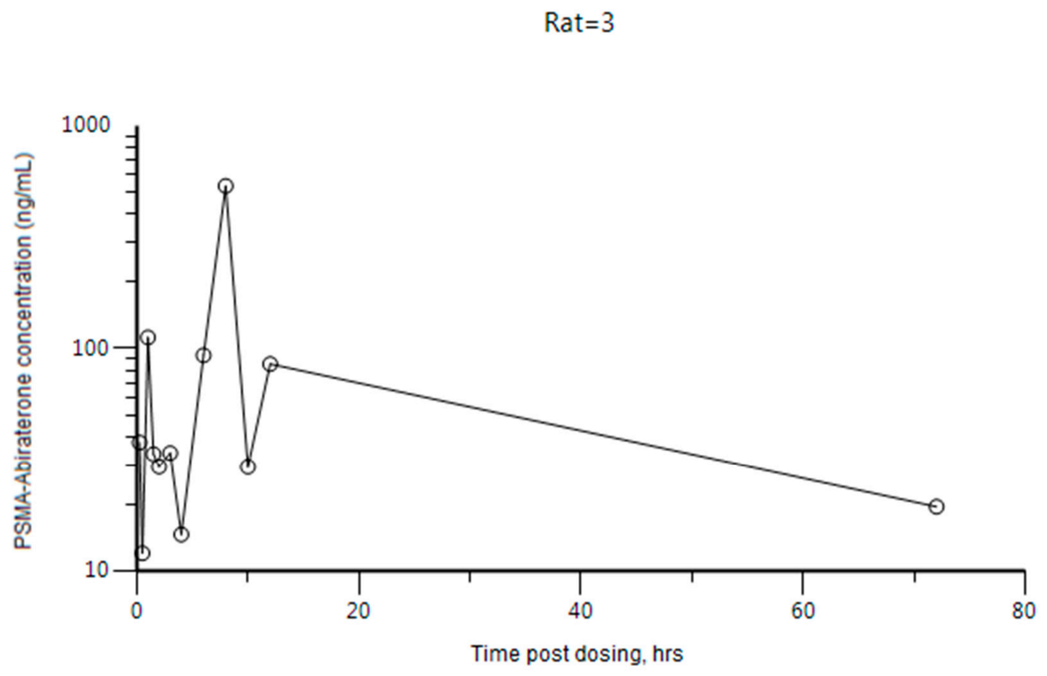
Rat=3
PSMA-Abiraterone concentration (ng/mL)
1000
100
10
0
20
40
60
80
Time post dosing, hrs

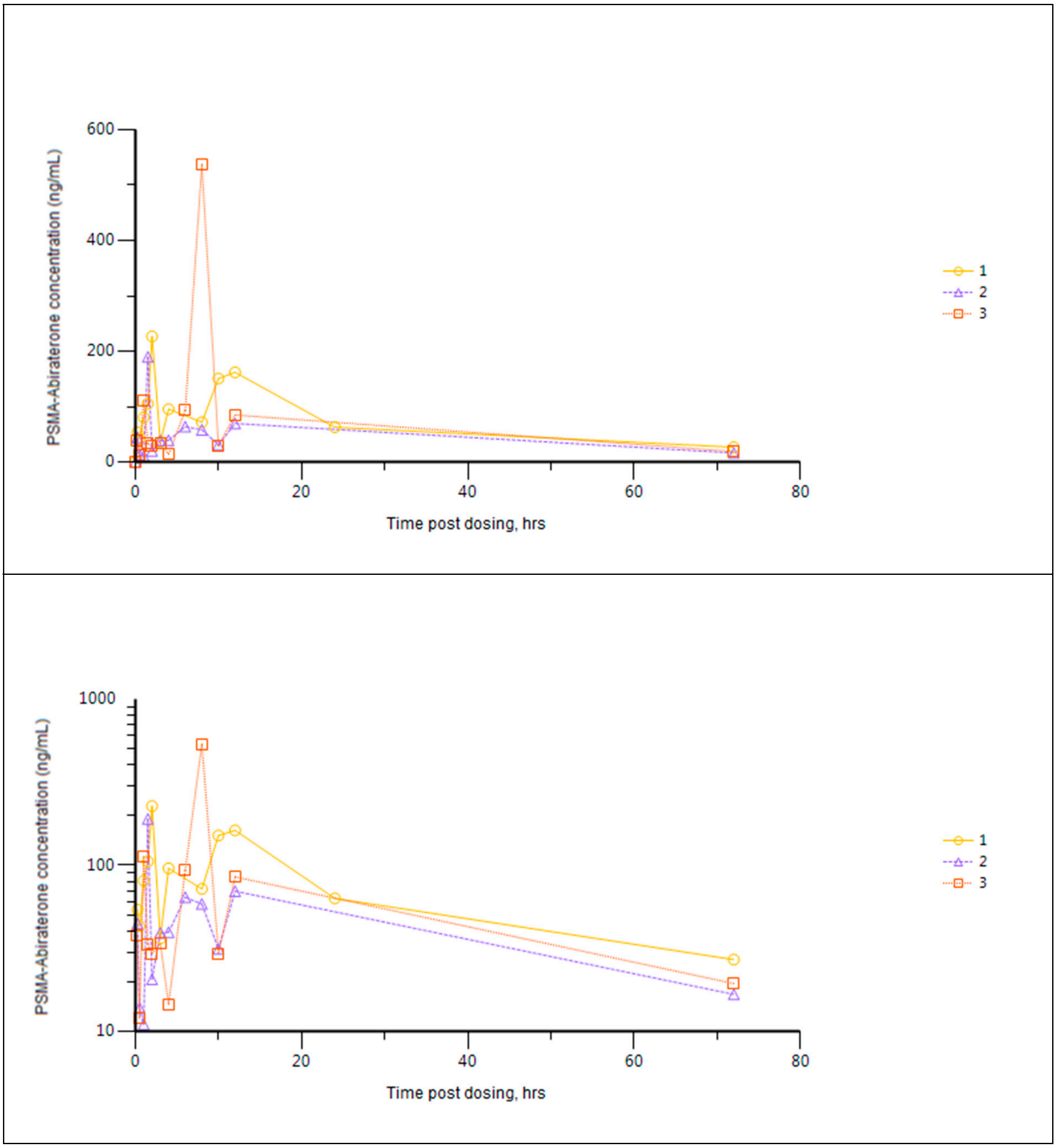

Figure S1. Pharmacokinetic curves for each rat (in linear and semi-logarithmic coordinates)

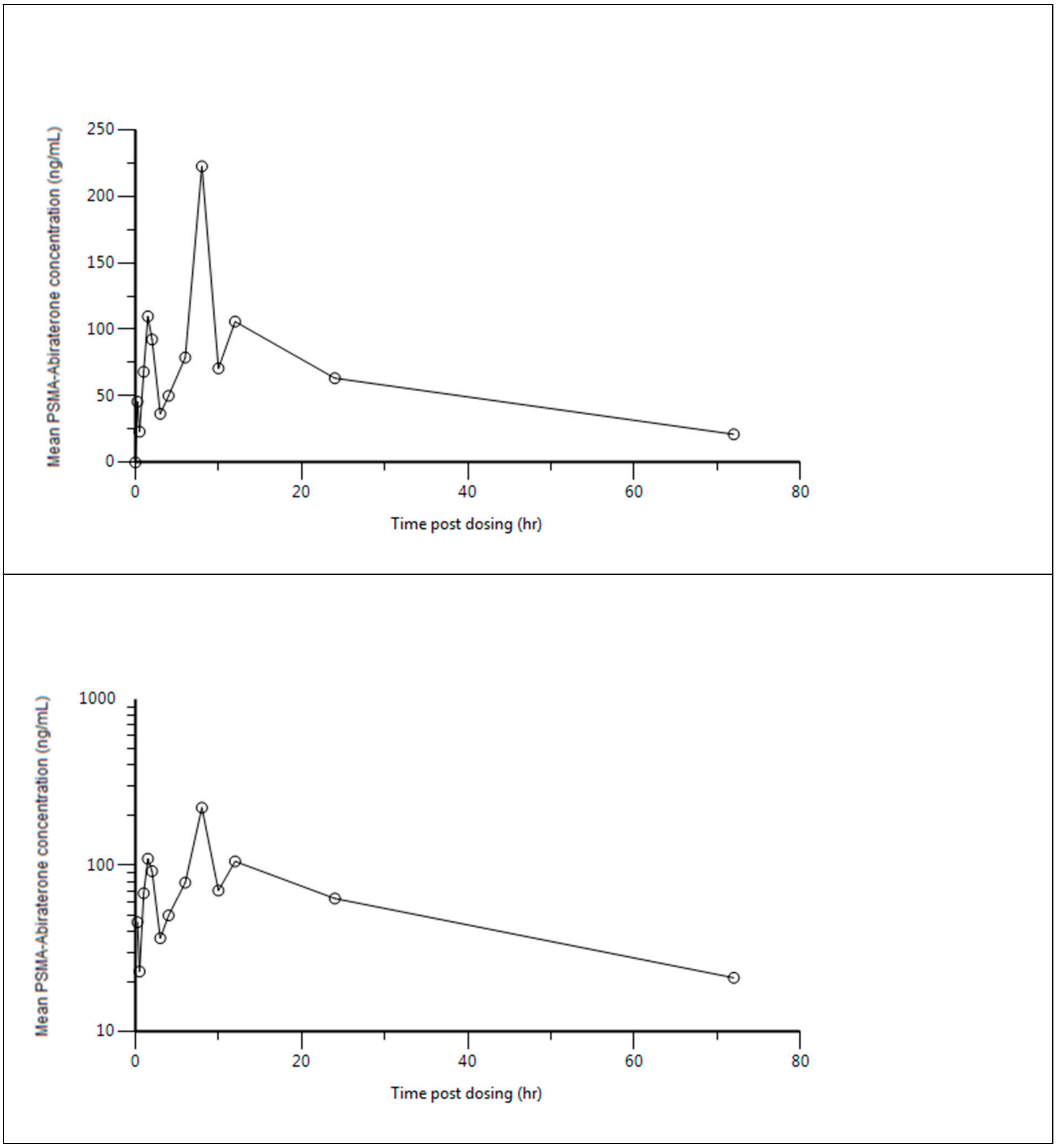

Figure S2. Average rat pharmacokinetic profiles (in linear and semilogarithmic coordinates)

Table S5. Pharmacokinetic parameters obtained on rats.

Rat	C_0 (μg/ml)	$AUC_{0\text{-}t}$ (h*μg/ml)	$AUC_{0\text{-}\infty}$ (h*μg/ml)	$AUC_{t\text{-}\infty}$/ $AUC_{0\text{-}\infty}$ (%)	K_{el} (1/h)	$T_{1/2}$ (h)	V_d (ml/kg)	Cl (ml/h/kg)	$MRT_{0\text{-}t}$ (h)
1	227.00	2.00	4529.62	5514.28	17.86	26.76	0.028	25.18	227.00
2	190.03	1.50	2824.63	3732.09	24.32	29.05	0.018	37.53	190.03
3	536.71	8.00	4020.19			25.69			536.71
**N**	**3**	**3**	**3**	**2**	**2**	**3**	**2**	**2**	**3**
**Mean**	**317.913**	**3.833**	**3791.480**	**4623.189**	**21.086**	**27.167**	**0.023**	**31.353**	**317.913**
**SD**	**190.383**	**3.617**	**875.200**	**1260.200**	**4.567**	**1.717**	**0.006**	**8.736**	**190.383**
**Min**	**190.03**	**1.50**	**2824.63**	**3732.09**	**17.86**	**25.69**	**0.018**	**25.18**	**190.03**
**Median**	**227.00**	**2.00**	**4020.19**	**4623.19**	**21.09**	**26.76**	**0.023**	**31.35**	**227.00**
**Max**	**536.71**	**8.00**	**4529.62**	**5514.28**	**24.32**	**29.05**	**0.028**	**37.53**	**536.71**
**Range**	**346.68**	**6.50**	**1704.99**	**1782.19**	**6.46**	**3.36**	**0.009**	**12.35**	**346.68**
**Geometric mean, %**	**59.9**	**94.4**	**23.1**	**27.3**	**21.7**	**6.3**	**27.9**	**27.9**	**59.9**
**N**	**285.012**	**2.884**	**3718.976**	**4536.499**	**20.837**	**27.131**	**0.023**	**30.738**	**285.012**

Table S6. Chromatography parameters in pharmacokinetic evaluation.

**Parameter**	Detalisation
**Column:**	Kromasil C18, 10x2.1 mm, 5 μm
**Thermostat temperature:**	50°C
**Elution:**	Gradient
**Mobile phase:**	Acetonitrile (B)

<table>
<tr><td></td><td>0,1% formic acid water solution (A)
<table>
<tr><td>Time, min.</td><td>A%</td><td>B%</td></tr>
<tr><td>0.00</td><td>70</td><td>30</td></tr>
<tr><td>2.00</td><td>5</td><td>95</td></tr>
<tr><td>2.01</td><td>70</td><td>30</td></tr>
<tr><td>5.00</td><td>70</td><td>30</td></tr>
</table></td></tr>
<tr><td>Flow rate:</td><td>0,5 ml/min</td></tr>
<tr><td>Sample volume:</td><td>10 μl</td></tr>
</table>

Cytochrome study

Study of the ability to inhibit the activity of human liver cytochromes (1A2, 2C8, 2C9, 2C19, 2D6, 3A4)

Stages of the research

1. Preparation of 0.05 M phosphate buffer, pH 7.4

1.1 1.74 g K_2HPO_4 were dissolved in 10 ml H_2O.

1.2 1.36 g KH_2PO_4 were dissolve in 10 ml H2O.

2. To prepare 0.05M buffer with pH=7.4:

2.1 8 mL of 1M K_2HPO_4 were combined with 2 mL of 1M KH_2PO_4.

2.2 The volume was Increased to 200 mL with H_2O.

2.3 The pH to 7.4 was adjust with 10-13 mL orthophosphoric acid.

2.4 The buffer was filtered. Keep it at +4ºC for up to three weeks.

3. Preparation of a suspension of human liver microsomes

A Starting solution of 20 mg/ml microsomes was diluted with 0.05 M phosphate buffer to 1.25 mg/ml (2.5x solution).

Table S7. Preparation of microsomes solution

	Final concentration	2.5 multiple concentration	Starting solution	Dilution factor	Volume starter solution for 4

					compounds, µL
HLM	0.5 mg/ml	1.25 mg/ml	20 mg/ml	16	775
0.05M phosphate buffer	50 mM	50 mM	50 mM		11625

*human liver microsomes

4. Preparation of the combined substrate solution.

4.1 The substrates were dissolved in acetonitrile with water, except for midazolam (2.8 mM in methanol) as shown in Table S20, and were mixed as shown in Table S21.

4.2 Then 934.2 µL of 0.05M phosphate buffer was added to the solution to prepare a 10-multiple substrate solution..

4.3 The final concentration of acetonitrile is 0.17% and methanol was 0.18%.

Table S8. Preparation of the Starting solution of substrates

Substrate	Molar weight	Starting solution (mM)	Solvent volume calculations for basic solutions, ml
Phenacetin	179.22	67 mM (33.8% acetonitrile)	Weight (mg) *1000/179.22/67
Testosterone	288.42	82 mM (80% acetonitrile)	Weight (mg) *1000/288.42/82
Tolbutamide	270.35	96 mM (50% acetonitrile)	Weight (mg) *1000/270.35/96
S-mephenytoin	218.25	50 mM (30% acetonitrile)	Weight (mg) *1000/218.25/50
Dextromethorphan hydrobromide	370.30	96 mM (10% acetonitrile)	Weight (mg) *1000/370.3/96

4.4 Starting solution of Dextromethorphan was further diluted by a factor of 10 with water to obtain a 9.6 mM concentration in 1% acetonitrile.

Table S9. Preparation of a combined solution of substrates.

CYP	Substrate	Final	10 multiple	Starting	Dilution	Volume

isoform		concentration, µM	concentration, mM	solution, mM	factor	starter solution for 1 ml, µL
1A2	Phenacetin	100	1	67	67	15
3A4	Midazolam	5	0.05	2.8	56	18
3A4	Testosterone	50	0.5	82	164	6
2C9	Tolbutamide	100	1	96	96	10.4
2C19	S-mephenytoin	30	0.3	50	166.7	6
2D6	Dextromethorphan	10	0.1	9.6	96	10.4

5. Preparation of solutions of test compounds

5.1 10 mM solutions were diluted in DMSO to 1 mM in acetonitrile/water (1:1).

5.2 6 serial dilutions were prepared in acetonitrile: water (1: 1) in increments of 3. The control inhibitors were: CYP1A2 for fluvoxamine, CYP2C9 for sulfophenazole, CYP2C19 for fluvoxamine, CYP2D6 for quinidine, and CYP3A4 for ketoconazole.

6. Preparation of a standard metabolite curve:

6.1. The metabolites were dissolved in acetonitrile with water or in water, except for 1-ONE-midazolam (2.9 mM in methanol) and 6β-OH-testosterone (0.33 mM in methanol) as indicated in Table S22, and were mixed as indicated in Table S23.

6.2 138.7 µl of 0.05 M buffer with 13.4 µl of acetonitrile were added to the mixture. Seven 10-fold calibration standards were prepared by serial dilution in steps of 2 in the solvent mixture (1.7% acetonitrile, 77.8% methanol in buffer).

Table S10. Preparation of starting solutions of metabolites.

Metabolite	Molar weight	Starting solution (mM)	Solvent volume calculations for basic solutions, ml
Paraxanthin	180.16	10 mM (10% acetonitrile)	Weight (mg) *1000/180.16/20
Acetaminophen	151.16	10 mM (H_2O)	Weight (mg) *1000/151.16/10
4-OH-tolbutamide	286.35	1 mM (1% acetonitrile)	Weight (mg) *1000/286.35
4-OH-mephenytoin	234.25	1 mM (1%	Weight (mg)

		acetonitrile)	*1000/234.25
Dextrorphan	257.34	6.7 mM (48% acetonitrile)	Weight (mg) *1000/257.24/6.7

Table S11. Preparation of combined solutions of metabolites.

Metabolite	Maximum concentration, μM	Minimum concentration, μM	10 multiple concentration, mM	Starting solution, mM	Dilution factor	Volume starter solution for buffer solution (1 ml), μL
Paraxanthin	10	0.156	0.1	10 mM (10% acetonitrile)	100	10
Acetaminophen	10	0.156	0.1	10 mM (H_2O)	100	10
4-OH-tolbutamide	3	0.0469	0.03	1 mM (1% acetonitrile)	33.3	30
4-OH-mephenytoin	1.5	0.0234	0.015	1 mM (1% acetonitrile)	66.67	15
Dextrorphan	3	0.0469	0.03	6.7 mM (48% acetonitrile)	223.3	4.5
1-OH-midazolam	50	0.7813	0.5	2.9 mM (methanol)	5.8	172.4
6β-OH-testosterone	20	0.3125	0.2	0.33 mM (methanol)	1.65	606

7. Preparation of the NANPR regeneration system

Table S12. Preparation of the NANPR regeneration system

	Final concentration	2 multiple concentration	Starting solution	Dilution factor	Volume for 1000 μL, (μL)

NANPR*	1.3 mM	2.6 mM	100 mM	38.5	26
Glucose-6-phosphate	3.3 mM	6.6 mM	300 mM	45.5	22
Glucose-6-phosphate dehydrogenase	1 mM	2 mM	200 mM	100	10
$MgCl_2$	3.3 mM	6.6 mM	260 mM	39.4	25.4
Buffer solution	50 mM	-	-	-	916.6

*NANPR - nicotinamide adenindine nucleotide phosphate reduced

8. Incubation

80 µl of microsomal solution (1.25/ml) were placed in wells of a 96-well polypropylene plate or 80 µl of buffer to "minimum" samples. 20 µl combined substrate solution (for experimental samples, maximum and minimum) or a 10-fold dilution of metabolites (for calibration samples) were added to each well, except for blank samples to which solvent buffer was added. Then 2 µl of test substances (or acetonitrile:water - 1:1 for maximum, minimum and calibration samples) were added to the appropriate wells. The solution was preincubated on a thermal shaker at 37oC at 400 rpm for 10 minutes. After preincubation 100 µl of cofactor solution were added to each well. After stirring, 160 µl from the "minimum" wells and calibration samples were transfered to 1.1 ml microtubes in a National Scientific plate. The proteins were precipitated by adding 400 µl of cold acetonitrile with 50 ng/ml propranololol, then the samples were mixed on a vortex and placed on ice for 15 minutes. Then the plate was centrifuged at 3000 rpm for 7 minutes. After adding cofactors, the reaction plate was incubated with the test substances and " maximums" on a thermal shaker for 30 minutes at 37oC at 400 rpm. Reaction was stoped in the same manner as for the calibration samples but with the addition of 2.9% methanol to acetonitrile-propranololol solution (50 ng/ml) for precipitation. 150 µl of supernatant were transfered to plates for LC/MS/MS analysis.

9. Calculations

The concentrations of the metabolites were calculated by using calibration curves of the chromatographic peak areas normalized to the signal of the internal standard (in the Analyst 1.5.2 software). Percent inhibition was estimated using the following equations:

% inhibition = 100 - (concentration experimental - concentration blank)/(concentration max - concentration min)*100,

concentration max - average concentration of the metabolite in the samples without inhibitor;

concentration blank - average concentration of the metabolite in samples with the substrate;

concentrate experimental - the average concentration of a metabolite with the compound under test or with an inhibitor.

IR_{50} - concentration of the compound at which 50% inhibition of cytochrome occurs

IR_{50} is calculated by regression of experimental data with a sigmoid curve using Graph Pad Prizm 5 software.

***In vitro* cytotoxicity**

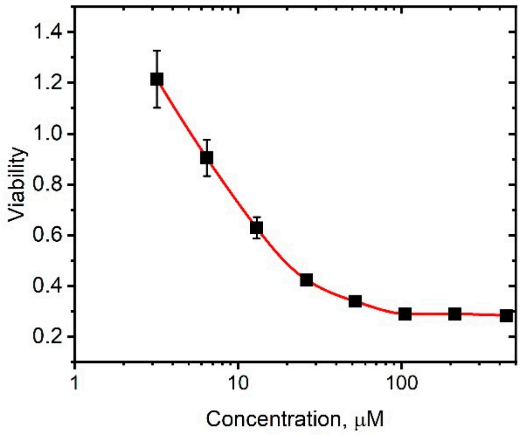

(A)

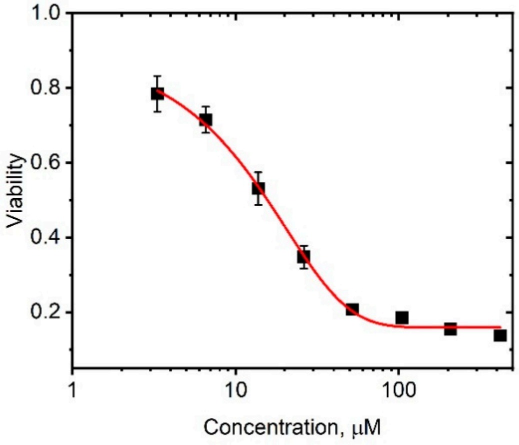

(B)

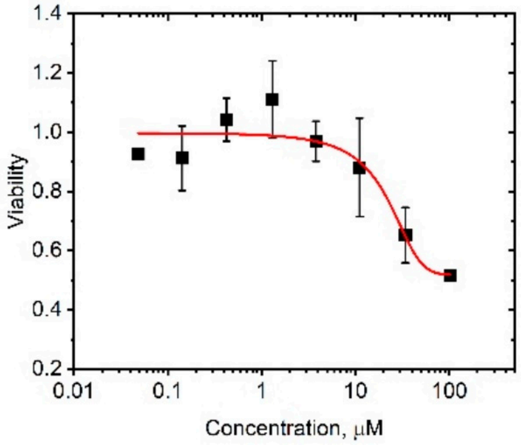

(C)

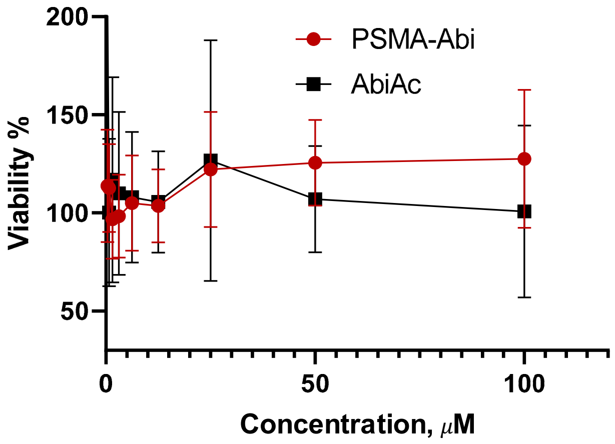

(D)

Figure S3. Cytotoxicity curves for PSMA-Abi on 22Rv1 (A) and PC-3 cells (B); Abi on 22Rv1 cells (C); PSMA-Abi and AbiAc on fibroblasts (D).

Cell cycle distribution

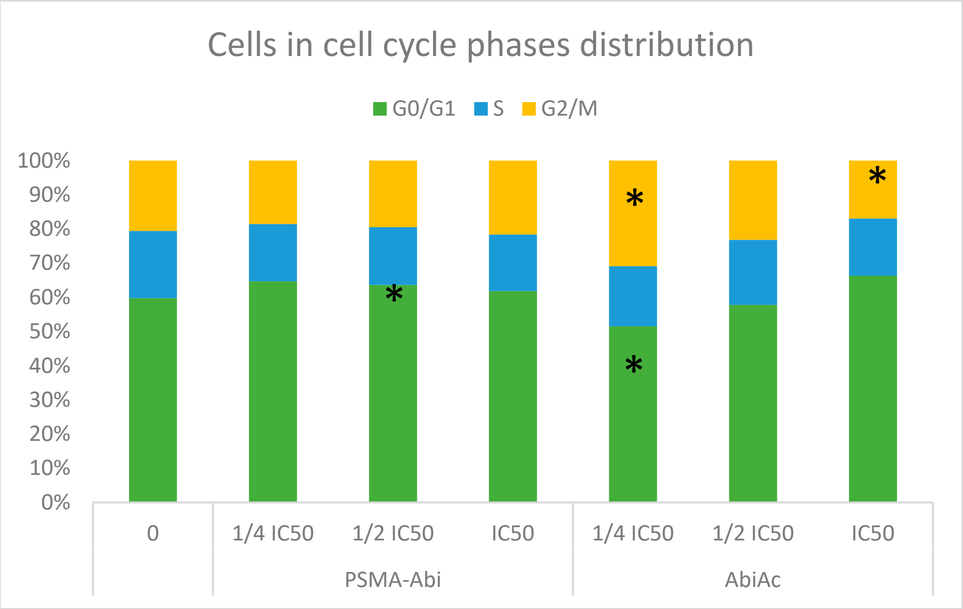

Figure S4. Cell cycle distribution obtained on 22Rv1 cells after incubation with **PSMA-Abi** and **AbiAc**.
